# Transcriptomic rewiring of the JAK–STAT pathway in circulating CD4^+^CLA^+^ and CD4^+^ naïve T cells from patients with atopic dermatitis and psoriasis

**DOI:** 10.3389/fimmu.2026.1782684

**Published:** 2026-04-20

**Authors:** Martin Pook, Regina Maruste, Peep Kolberg, Kaur Alasoo, Tõnis Org, Liisi Raam, Anu Remm, Dario Greco, Antonio Federico, Külli Kingo, Kai Kisand, Ana Rebane

**Affiliations:** 1 Institute of Biomedicine and Translational Medicine, Faculty of Medicine, University of Tartu, Tartu, Estonia; 2 Institute of Computer Science, Faculty of Science and Technology, University of Tartu, Tartu, Estonia; 3 Institute of Molecular and Cell Biology, Faculty of Science and Technology, University of Tartu, Tartu, Estonia; 4 Institute of Genomics, Faculty of Science and Technology, University of Tartu, Tartu, Estonia; 5 Institute of Clinical Medicine, Faculty of Medicine, University of Tartu, Tartu, Estonia; 6 Clinic of Dermatology, Tartu University Hospital, Tartu, Estonia; 7 Finnish Hub for Development and Validation of Integrated Approaches (FHAIVE), Faculty of Medicine and Health Technology, Tampere University, Tampere, Finland; 8 Division of Pharmaceutical Biosciences, Faculty of Pharmacy, University of Helsinki, Helsinki, Finland; 9 Institute of Biotechnology, University of Helsinki, Helsinki, Finland

**Keywords:** ATAC sequencing, atopic dermatitis, CD4^+^ T cells, cutaneous lymphocyte-associated antigen, psoriasis

## Abstract

**Background:**

Skin-homing cutaneous lymphocyte-associated antigen (CLA)-expressing T cells play a key role in the pathogenesis of atopic dermatitis (AD) and psoriasis (Ps). We aimed to characterize the transcriptomic and epigenetic profiles of circulating CD4^+^CLA^+^ and CD4^+^ naïve T cells from patients with AD and Ps to find shared and unique molecular signatures associated with the diseases.

**Methods:**

Circulating CD4^+^CLA^+^ and CD4^+^ naïve T cells were sorted from the peripheral blood mononuclear cells of patients with AD (*n* = 11), those with Ps (*n* = 10), and healthy individuals (*n* = 11), followed by assay for transposase-accessible chromatin sequencing (ATAC-seq) and mRNA sequencing and data analyses.

**Results:**

Transcriptomic and epigenetic landscapes differed markedly between CD4^+^CLA^+^ and CD4^+^ naïve T cells. In both AD and Ps, transcriptomic alterations within these cell populations were substantial, whereas changes in chromatin accessibility were relatively modest. In CLA^+^ T cells, patients with AD and Ps exhibited altered expression of genes involved in T-cell activation, cell cycle, and JAK–STAT signaling. These effects were more pronounced in AD, while a stronger association with innate immune activation was seen in Ps. Notably, CD4^+^ naïve T cells also exhibited disease-associated transcriptomic changes in both AD and Ps, including alterations in the JAK–STAT pathway and changes in the expression of IL-2 receptor components. Epigenetic profiling further revealed disease-associated chromatin regions linked to transcription factors involved in immune regulation.

**Conclusion:**

Both CD4^+^CLA^+^ and CD4^+^ naïve T cells exhibit transcriptomic and epigenetic alterations in AD and Ps, suggesting the influence of the chronic inflammatory milieu leading to shared and disease-specific changes, including transcriptomic rewiring of the JAK–STAT pathway in both diseases.

## 1 Introduction

Atopic dermatitis (AD) and psoriasis (Ps) are chronic inflammatory skin diseases, in which the persistent inflammatory milieu is associated with dysregulated T-cell responses (1). While AD is primarily associated with increased T helper (Th)2-driven inflammation and Ps is associated with Th17-mediated immunity, Th1, Th22, and Th17 and innate immune pathways may additionally influence both conditions (2–5). In lesional skin, both diseases share striking similarities in gene expression signatures, yet each also exhibits distinct disease-specific alterations, with AD displaying greater molecular heterogeneity than Ps (6, 7). Altered T-cell responses in AD and Ps are strongly, though not exclusively, associated with circulating CD4^+^ memory T cells expressing cutaneous lymphocyte-associated antigen (CLA), which enables their homing to the skin (8, 9). Therefore, at least a subset of circulating CD4^+^CLA^+^ T cells is influenced by inflammatory events in the skin and may serve as valuable marker cells for AD and Ps.

CD4^+^CLA^+^ T cells consist almost exclusively of memory T cells capable of infiltrating cutaneous tissue, with very limited location to other tissues or lymphoid organs (10, 11). Traditionally, memory T cells are classified into two main subsets based on CCR7 expression: central memory T cells (CCR7^+^, T_CM_) and effector memory T cells (CCR7^−^, T_EM_) (12). However, CCR7 expression appears to have little impact on the egress of CD4^+^ T cells from inflamed skin (13). Both the T_CM_ and T_EM_ are part of the circulating CD4^+^CLA^+^ T-cell population along with the T regulatory (Treg) cells (14). Notably, tissue-resident memory T cells (T_RM_) can also contribute to the circulating population (15). To date, CD4^+^CLA^+^ T cells have been predominantly characterized in terms of phenotype and function, including transcriptomic profiling. However, previous epigenetic studies in AD have focused on DNA methylation (16), while alterations at the chromatin level have not yet been addressed in either AD or Ps. Given the translational significance (17), a deeper understanding of how CD4^+^CLA^+^ T cells can be used as marker cells in AD and Ps is needed.

In this study, we used assay for transposase-accessible chromatin sequencing (ATAC-seq) and RNA sequencing (RNA-seq) for the analysis of circulating CD4^+^CLA^+^ T cells and CD4^+^ naïve T cells from peripheral blood of patients with AD, those with Ps, and healthy controls (HC) with the aim of identifying disease-specific and shared epigenetic and transcriptomic signatures and signaling pathways in both diseases.

## 2 Materials and methods

### 2.1 Patient cohort and overview of the experimental design

Whole blood samples from 11 patients with AD, 10 patients with Ps (plaque psoriasis), and 11 HC were collected at Tartu University Hospital (**Supplementary Tables E1**, **E2**), followed by same-day isolation of peripheral blood mononuclear cells (PBMCs). All patients were with moderate to severe disease severity status and treatment-free for at least 2 weeks prior to recruitment. Disease and treatment characteristics are provided in **Supplementary Table E3**. None of the patients had prior exposure to biologics.

**Figure 1:**
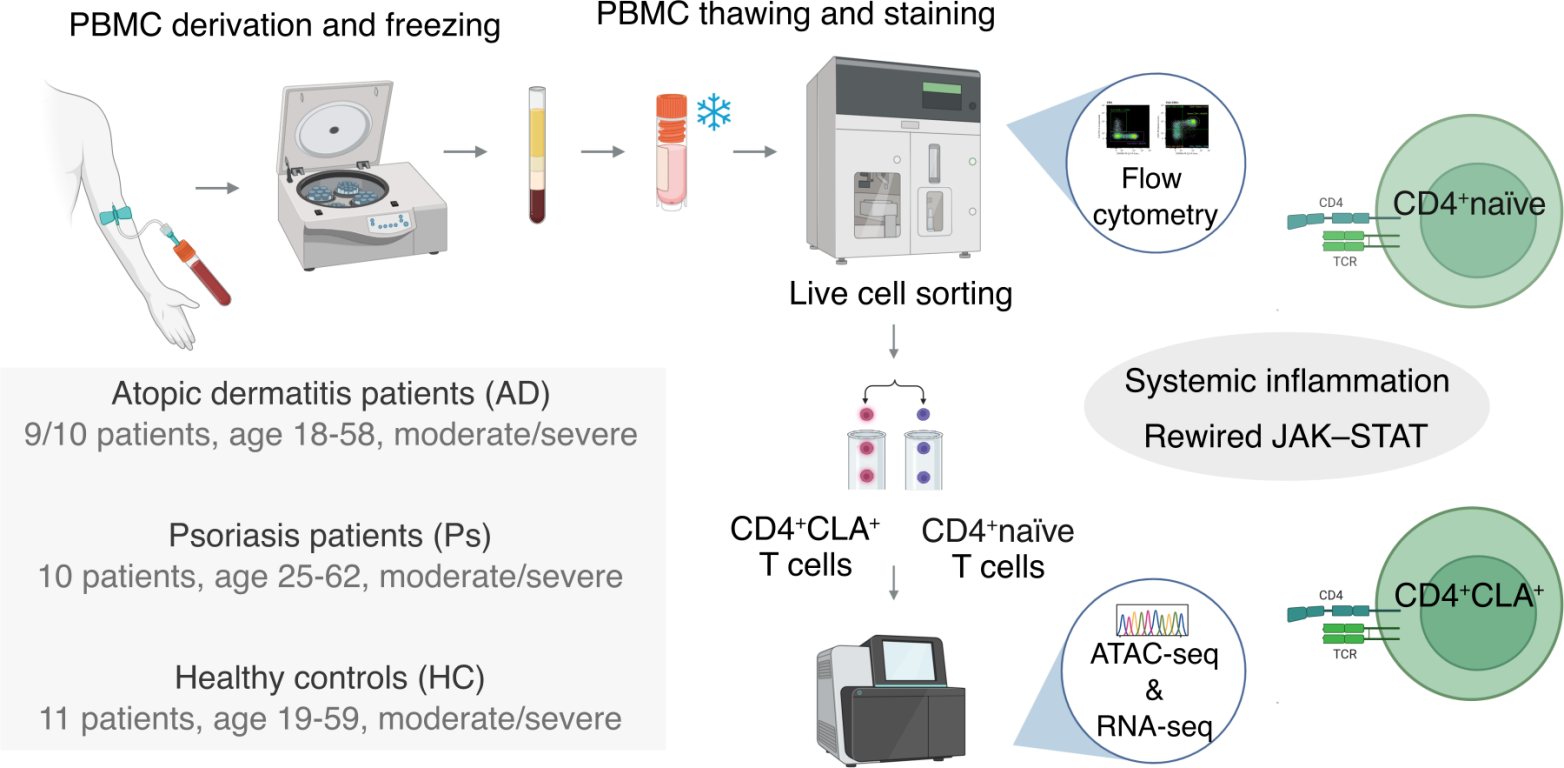
Graphical abstract of the study. Transcriptomic and epigenetic landscapes of CD4^+^CLA^+^ and naïve T cells in AD and Ps. Human peripheral blood mononuclear cells were isolated from patients with moderate-to-severe AD and Ps, as well as from healthy control individuals, and cryopreserved until flow cytometry and cell sorting. Subsequent ATAC-seq and RNA-seq analyses were performed to characterize the epigenetic and transcriptomic landscapes. These analyses indicated systemic inflammation affecting both cell types and suggested transcriptomic rewiring of the JAK–STAT pathway in these diseases. Created with BioRender.com, https://BioRender.com/c03w502.

PBMCs were cryopreserved until immunostaining with fluorochrome-conjugated antibodies (**Supplementary Table E4**) and sorted into CD4^+^CLA^+^ and CD4^+^ naïve T-cell populations using an LE-MA900FP (Sony) sorter with settings provided in **Supplementary Tables E5**, **E6** followed by ATAC-seq and RNA-seq protocols and data analysis as overviewed in **Figure 1**.

### 2.2 Blood sample acquisition by venipuncture

Venous blood (20 mL) was asked to be donated from patients and healthy volunteers after obtaining informed consent. Blood samples were collected into vacutainer EDTA tubes (Becton, Dickinson and Company), kept at room temperature, and used for PBMC separation within 4 h after collection.

### 2.3 Purification and freezing of PBMCs

Purification of PBMCs was carried out under BSL2 hood. Vacutainer tubes were first centrifuged (300 × *g*, 10 min, room temperature, high acceleration, no brake) for plasma separation. The remaining blood sample was diluted with 21 mL of PBS (w/o Ca and Mg, room temperature). Diluted blood (20 mL) was used per 10 mL of Ficoll solution for subsequent density gradient centrifugation on Ficoll (400 × *g*, 30 min, 20 °C, low acceleration, no brake). Lymphocyte fraction was transferred into new 50-mL polypropylene tubes and diluted by gentle suspending with PBS (w/o Ca and Mg, room temperature) obtaining a final volume of 45 mL. Cells were pelleted by centrifugation (400 × *g*, 10 min, 20 °C, high acceleration, high brake), and the supernatant was removed. Thereafter, cells were further washed two times with 50 mL of PBS (w/o Ca and Mg, 4 °C) followed by centrifugation (300 × *g*, 10 min, 20°C, high acceleration, high brake). Cells were frozen with the CTL-Cryo ABC Media Kit (Cellular Technology Limited) according to the manufacturer’s protocol with adjustment for cell concentration (10^7^ cells per 500 µL CTL-Cryo C) and stored at −150 °C.

### 2.4 Flow cytometry and cell sorting

PBMCs were thawed with warm (37 °C) RPMI 1640 medium (Thermo Fisher Scientific) supplemented with 10% heat-inactivated fetal bovine serum, penicillin 100 IU/mL and streptomycin 100 μg/mL and pelleted by centrifugation (200 × *g*, 10 min, room temperature) for subsequent resuspending in 10 mL of running buffer (RB, 5% bovine serum albumin, 2 mM EDTA in PBS) at 4 °C. From here on, the cells were always kept at 4 °C. Cells were counted with a cell counter (Luna-FL, Logos Biosystems) and pelleted by centrifugation (300 × *g*, 10 min). A total of 10^7^ cells were resuspended in 100 µL of antibody staining mix in RB (**Table E4**) and incubated 30 min in the dark. Cells were washed once with 1 mL of RB and pelleted by centrifugation (400 × *g*, 5 min). Cell pellet was resuspended in 400 µL of RB and filtered through a 40-µm strainer for subsequent cell sorting (collection at 4 °C) into BSA-coated 5-mL polystyrene tubes (FC tubes). 7AAD (BioLegend, final concentration, 0.5 µL/mL) was added just before cell sorting for live/dead cell discrimination. The gating strategy was based on using only single (FSC-A vs. FSC-H) viable (7AAD^−^) cells for subsequent analysis. Firstly, T cells were gated expressing CD3 (CD3^+^) and CD4^+^ T cells were further selected based on the expression CD4 and CD8 (CD4^+^CD8^−^). From the latter, CLA^+^CD4^+^ T cells were selected based on the expression of CLA and CD45RA (CLA^+^CD45RA^−^). CD4^+^ naïve T cells were identified expressing CCR7 and CD45RA (CCR7^+^CD45RA^+^) and were selected from CLA-negative CD4^+^ T cells (CD3^+^CD4^+^CD8^−^CLA^−^). To analyze proportions of Treg cells from CD4^+^CLA^+^ T cells, cells were defined based on the expression of CD127 and CD25 (CD127^−^CD25^+^) and selected from CD4^+^CLA^+^ T cells (CD^+^CD4^+^CD8^−^CLA^+^CD45RA^−^). The latter population was also analyzed for the expression of CCR7 to estimate the proportions of central memory T cells (CCR7^+^) and effector memory T cells (CCR7^−^). The proportion of naïve Treg cells under CD4^+^ naïve T cells was measured based on the expression of CD127 and CD25 (CD127^−^CD25^+^). Boolean gates (AND, NOT) were used to select CLA^+^CD4^+^ or CD4^+^ naïve T cells without Tregs. The latter populations were analyzed for proportions of CD25^+^ cells. Median fluorescence intensity (MFI) was used to measure expression levels of CD25 and CCR7 in selected gates. The gates and parameter values (**Supplementary Figure E16**, **Supplementary Tables E5**, **E6**) were set using FMO controls, and cells were analyzed and sorted with LE-MA900FP (Sony) into FC tubes preloaded with 100 µL of resuspension buffer (RSB; 10 mM Tris-HCl, pH 7.4, 10 mM NaCl, and 3 mM MgCl_2_ in water) for ATAC-seq and into 500 µL of QIAzol (QIagen) for RNA-seq. Technical details for sorting are described in **Tables E5** and **E6**. A total of 50,000 sorted cells were collected for ATAC-seq, while for RNA-seq, on average 100,000 CD4^+^ naïve cells and 50,000 CD4^+^CLA^+^ T cells were sorted per sample.

### 2.5 ATAC sequencing and data analysis

Quick centrifugation (300 × *g*, 15 s, 4 °C) was applied to the FC tubes with sorted material in order to collect all cell suspension drops. Cell suspension was transferred into 1.5-mL DNA LoBind tubes, and FC tubes were washed with 1 mL of RSB to get residual cells. Cells were pelleted (500 × *g*, 5 min, 4 °C) and gently resuspended (by pipetting up and down three times) in 50 µL of ice-cold lysis buffer (10 mM Tris-HCl, pH 7.4, 10 mM NaCl, 3 mM MgCl_2_, and 0.1% IGEPAL CA-630). Immediately thereafter, 1 mL of ice-cold RSB was added to the 50-µL lysis suspension and samples were centrifuged (500 × *g*, 10 min, 4 °C). Supernatant was gently removed, and tubes with cell pellet were put on ice for subsequent Tn5 transposase treatment with Illumina Tagment DNA Enzyme (TDE1) and Buffer (TD). Pellet was resuspended (by pipetting up and down six times) in 50 µL of transposase treatment solution (25 µL of TD + 2.5 µL of TDE1 + 22.5 µL of nuclease-free water) and incubated at 37 °C with 1,000 rpm rotation (ThermoMixer C, Eppendorf) for 30 min. Immediately thereafter, DNA purification was performed (DNA Clean and Concentrator-5 Kit, Zymo). Purified DNA was stored at −20 °C until proceeding with all the analysis samples for subsequent PCR amplification and preparation of sequencing libraries. PCR amplification (five PCR cycles) and sequencing libraries were prepared with unique dual indexes using the Illumina DNA Prep kit (Illumina) similarly as described before (18). Quality control of the libraries and sequencing (150 bp paired-end, 25M reads, Illumina Novoseq) were performed by Novogene.

Analysis of ATAC-seq data, from raw sequencing reads to identification of chromatin accessibility regions (ChARs) and quantification, was performed using the nf-core/atacseq v1.2.1 pipeline using hg38 as the reference genome, calling narrow peaks and using default parameters for other settings. Peaks were annotated with GREAT (19) (version 4.0.4, single nearest gene rule 1000 kb). Principal component analysis (PCA) of VST-normalized (DEseq2 output from nf-core/atacseq) from the top 500 most variable gene counts was performed using scikit-learn (data scaling: sklearn_data_preprocess, Galaxy Version 1.0.8.2; sklearn_pca, Galaxy Version 1.0.8.4+galaxy0). Volcano Plot (Galaxy Version 0.0.3) was used to plot annotated differential accessibility regions. Analysis of enrichment of transcription factor (TF) binding sites was performed with WhichTF (20) using a Base-mean count of at least 15, filtering for input. Main output data tables from the ATAC-seq analysis are provided under **Supplementary Data Tables**. Examples of ATAC-seq coverage and peak detection can be found in **Supplementary Figures E17-E18**. Patient sensitive raw sequencing data are deposited into ELIXIR Luxembourg Data Catalogue (b70ca632-813e-11ed-9414-acde48001122) and can be made available upon request.

### 2.6 mRNA sequencing and data analysis

RNA was purified using the miRNeasy Micro Kit (Qiagen) and on-column DNase treatment with RNase-Free DNase Set (Qiagen). Library preparation with poly-A, enrichment with SMARTer Ultra Low Input RNA Kit (Clontech Laboratories), and sequencing (150 bp paired-end, 20M reads) on the Novoseq 6000 platform (Illumina) were carried out by Macrogen.

Data analysis was performed using Galaxy platform tools. FASTQ files of sequencing data were preprocessed with “fastp” (Galaxy Version 0.23.2+galaxy0) using default settings with additional filtering parameters “qualified quality phred 20” and “length required 20”. Mapping against hg38 Canonical was done using RNA STAR (Galaxy Version 2.7.8a+galaxy1) and NCBI RefSeq gene model with default parameters and using ReadLength-1 parameter for constructing the splice junctions database. Featurecounts (Galaxy Version 2.0.1) was used for counting reads as fragments. Differential expression (DE) analysis was performed with DESeq2 (Galaxy Version 2.11.40.6+galaxy1) using one factor with six factor levels and correction for age (centered, scaled) as a continuous covariate. DE results having a Base-mean count of at least 15 were selected for further analysis. A *p*-adjusted threshold of 0.05 was used to select DE genes. PCA of VST-normalized (DEseq2 output) from the top 500 most variable gene counts was performed using scikit-learn (data scaling: sklearn_data_preprocess, Galaxy Version 1.0.8.2; sklearn_pca, Galaxy Version 1.0.8.4+galaxy0). Volcano Plot (Galaxy Version 0.0.3) was used to plot DE-expressed genes. Pheatmap library (1.0.12) was used for creating heatmaps in R (DESeq2 VST-normalized reads, scaled on rows). Functional enrichment analyses were performed using the STRING database web interface (version 12.0, https://string-db.org/). IPA Core Analysis (Ingenuity Systems Inc.) was performed, and Graphical Summary was used as an output to illustrate the most significantly activated or inhibited upstream regulators, diseases, functions, and pathways. Main output data tables from the RNA-seq analysis are provided under **Supplementary Data Tables**. Patient sensitive raw sequencing data are deposited into ELIXIR Luxembourg Data Catalogue (b70ca632-813e-11ed-9414-acde48001122) and can be made available upon request.

### 2.7 Statistics

Kruskal–Wallis and Dunn’s multiple comparison test was used to compare proportions (%) of cell populations and MFI values from flow cytometry data by using GraphPad Prism (10.1.0). All other statistical analyses were performed as described above with the dedicated bioinformatic tools (DESeq2, STRING web-based tool, and WhichTF).

## 3 Results

### 3.1 Flow cytometric analysis of CD4^+^CLA^+^ T cells reveals differences between AD and Ps in the proportions of T_CM_ cells and Treg subpopulations

**Figure 2:**
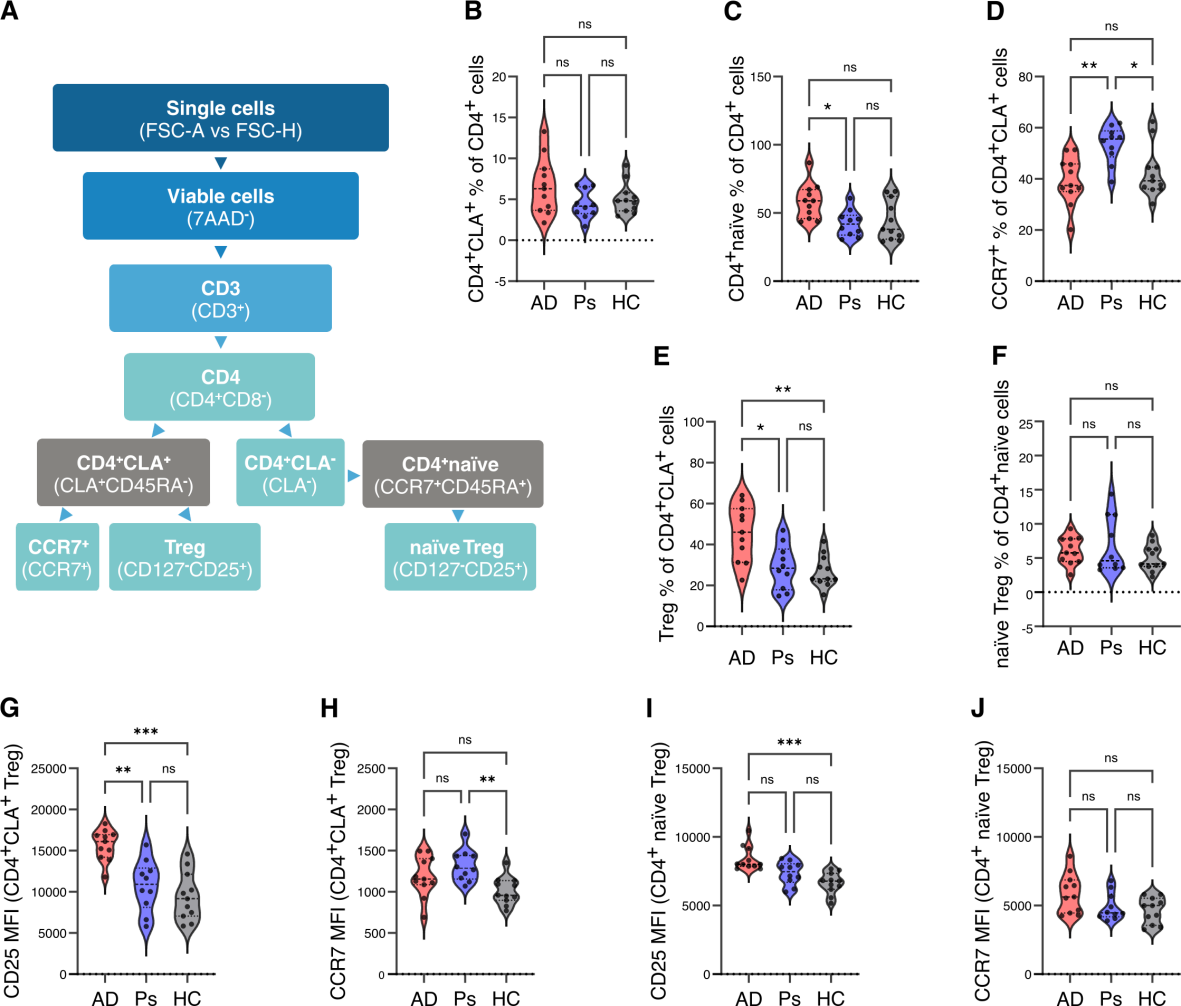
Cell sorting strategy of CD4^+^CLA^+^ T cells (CLA) and CD4^+^ naïve (naïve) T cells for ATAC-seq and RNA-seq analysis. **(A)** Overview of gating strategy for cell sorting and subpopulation analyses. Sorted cell populations are in gray boxes. Proportions (%) of CD4^+^CLA^+^ T cells **(B)** and CD4^+^ naïve T cells **(C)** among CD4^+^ T cells, CCR7^+^ T cells among CD4^+^CLA^+^ T cells **(D)**, Treg cells among CD4^+^CLA^+^ T cells **(E)**, and naïve Treg cells **(F)** among CD4^+^ naïve T cells. Median fluorescence intensity (MFI) of CD25 **(G)** or CCR7 **(H)** from CD4^+^CLA^+^ Treg cells and CD25 **(I)** or CCR7 **(J)** from CD4^+^ naïve Treg cells in AD, Ps, and HC. Statistical differences in proportions (%) of cell populations or MFI are compared with Kruskal–Wallis and Dunn’s multiple comparison test (* p-adjusted <= 0.05; ** p-adjusted <= 0.01; *** p-adjusted <= 0.001).

To perform transcriptome and epigenetic analysis of CD4^+^CLA^+^ T-cell populations in AD and Ps, a broad population of CD4^+^ T cells expressing CLA (CD4^+^CLA^+^) was gated (**Figure 2A**, **Supplementary Figure E16**). CD4^+^ naïve T cells were selected (**Figure 2A**, **Supplementary Figure E16**) for comparison as control cells circulating in the blood and supposedly not influenced by tissue-specific cues. Subsequently, flow cytometric analysis and sorting of both populations (**Figure 2A**) were performed. No significant differences in proportions of viable cells between samples from AD, Ps, or HC was seen, while in general, less CD4^+^CLA^+^ (average 2%) compared to CD4^+^ naïve T cells (average 19%) were among live PBMCs (**Supplementary Figures E1A, B**). When comparing the sub-populations of CD4^+^ T cells between AD and Ps groups (**Figures 2B, C**), there was an increase in CD4^+^ naïve T cells in AD (average 58%) compared to Ps (average 42%). Flow cytometric analysis of CCR7 expression of CD4^+^CLA^+^ T cells suggested that there were more T_CM_ cells (CCR7^+^) in Ps (**Figure 2D**). In addition, there were more Treg cells in case of AD among the CD4^+^CLA^+^ T cells compared to Ps or HC sample groups (**Figure 2E**), while there were no differences between naïve Treg cell proportions among CD4^+^ naïve T cells (**Figure 2F**). Further analysis of CD4^+^CLA^+^ Tregs and CD4^+^ naïve Tregs showed that CD25 is expressed at a higher level in AD in both cell types (**Figures 2G, I**, respectively), while CCR7 is expressed at a higher level in Ps CD4^+^CLA^+^ Tregs (**Figure 2H**) but not in CD4^+^ naïve Tregs (**Figure 2J**). At the same time, no differences were detected in CD25^+^ cells when analyzed in CD4^+^CLA^+^ or CD4^+^ naïve Treg population without Tregs (**Supplementary Figures E2**, **E3**, respectively).

### 3.2 Differences in transcriptome of CD4^+^CLA^+^ and naïve T cells are more pronounced than those in chromatin accessibility in AD and Ps

Next, sorted CD4^+^CLA^+^ and CD4^+^ naïve T-cell populations were subjected to ATAC-seq and RNA-seq analyses, followed by quantitative identification of chromatin accessibility regions (ChARs) and differentially expressed genes (DEGs).

**Figure 3:**
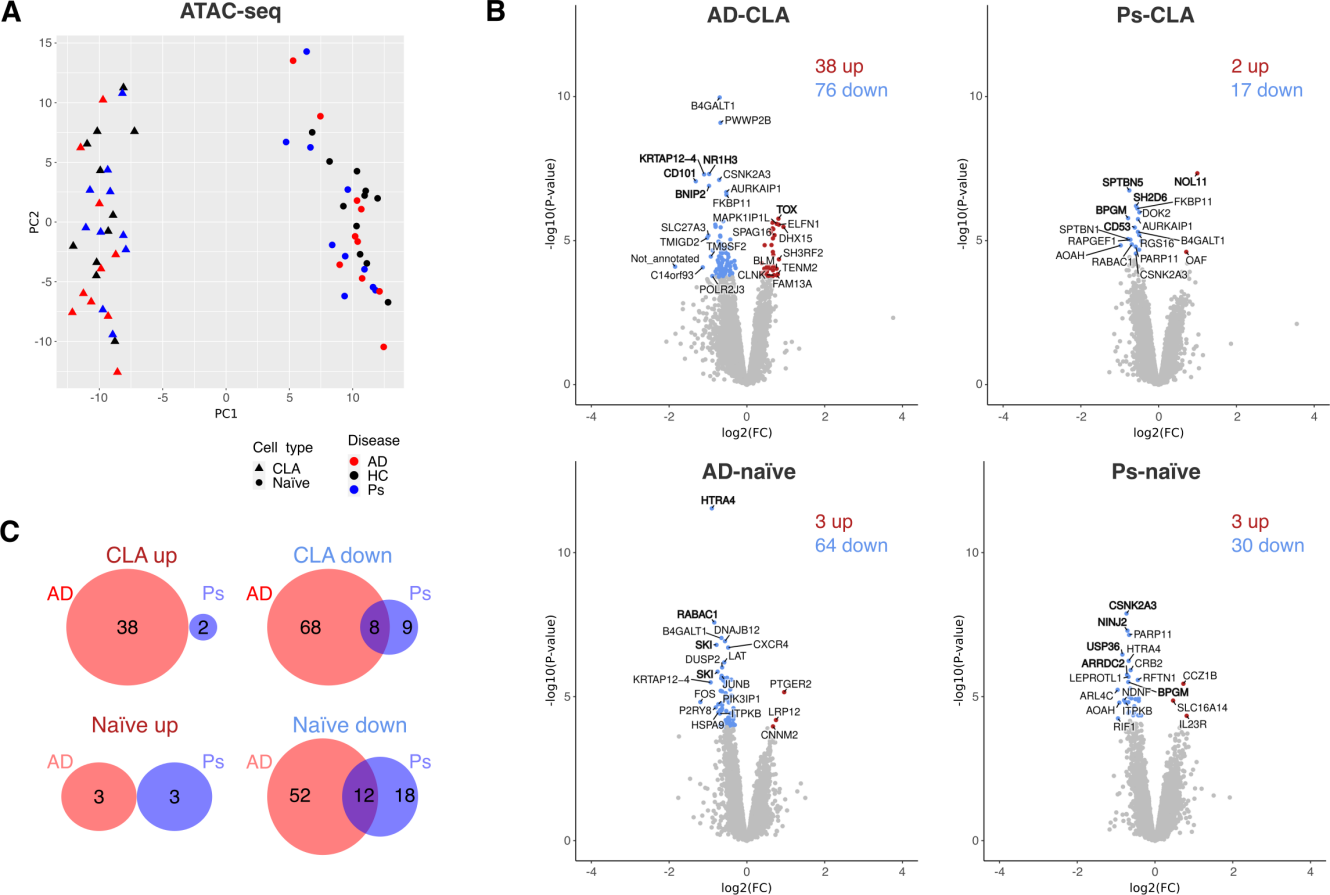
ATAC-seq analysis of CD4^+^CLA^+^ (CLA) and CD4^+^ naïve (naïve) T cells shows relatively few disease-linked differences in accessibility of the chromatin. **(A)** Principal component analysis of VST-normalized counts from ATAC-seq. **(B)** Volcano plots of more open (up, red color) and closed (down, blue color) chromatin areas (*p*-adj < 0.05) are presented compared with HC. The peaks among the top 10 most up and the top 10 most down based on FC are presented with annotations (GREAT, single nearest gene, 1,000 kb) while annotations in bold are among the top 10 most significant differences based on adjusted *p*-value. **(C)** Venn diagrams of shared and disease-specific up- or downregulated chromatin regions (*p*-adj < 0.05).

**Figure 4:**
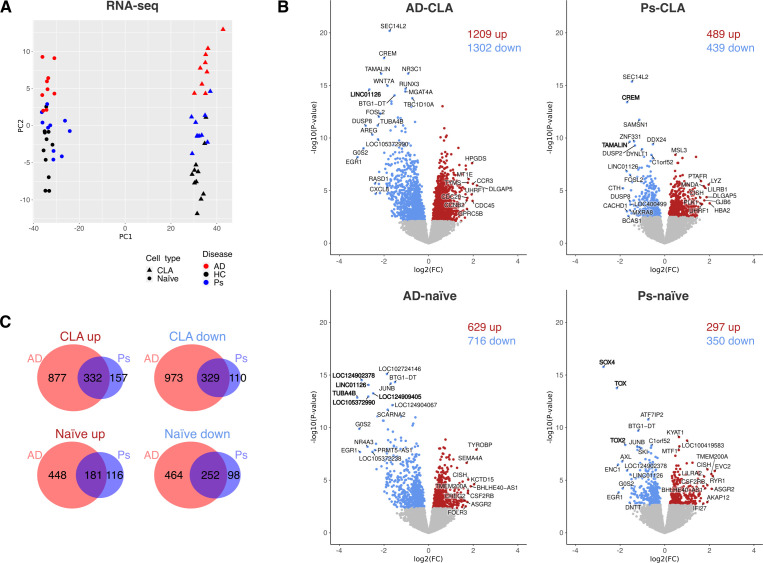
RNA-seq analysis of CD4^+^CLA^+^ (CLA) and CD4^+^ naïve (naïve) T cells highlights differences between AD and Ps. **(A)** Principal component analysis of VST-normalized counts from RNA-seq. **(B)** Volcano blots of DEGs significantly (*p*-adj < 0.05) upregulated (up, red color) or downregulated (down, blue color) compared with HC. **(C)** Venn diagrams of shared and disease-specific DEGs being up- or downregulated (*p*-adj < 0.05).

First, PCA was performed on ATAC-seq (**Figure 3A**) and RNA-seq (**Figure 4A**) normalized read counts, which revealed clear distinctions between CD4^+^CLA^+^ and CD4^+^ naïve T cells. The overall differences in transcriptomic profiles within different cell types showed the strongest segregation for CD4^+^CLA^+^ T cells, with AD segregating more than Ps from the HC group (**Figure 4A**). ATAC-seq did not reveal major changes between the disease and the HC group (**Figure 3A**), while still a few statistically different ChARs (**Figure 3B**) were detected. Overall, there were more downregulated than upregulated ChARs and DEGs in both cell types and diseases compared to the HC group, except for DEGs in the CD4^+^CLA^+^ T cells from patients with Ps (**Figure 4B**). The most significant changes were also detected among downregulated ChARs (**Figure 3B**) and DEGs (**Figure 4B**). When comparing disease groups (**Figures 3B**, **4B**), AD had more DEGs among both CD4^+^CLA^+^ and CD4^+^ naïve T cells (AD-CLA: 2511; AD-naïve: 1345) and differences in the accessible chromatin regions (AD-CLA: 114; AD-naïve: 67) than Ps (DEGs, Ps-CLA: 928, Ps-naïve: 647; ChAR, Ps-CLA: 19, Ps-naïve: 33).

Next, the ATAC-seq and RNA-seq results were analyzed to identify disease-specific ChARs and DEGs and those shared between AD and Ps. Shared ChARs (**Figure 3C**) were identified exclusively among the downregulated regions (CLA 8; naïve: 12), which was expected due to the limited number of upregulated regions in the Ps, while shared DEGs (**Figure 4C**) were detected among up- and downregulated genes (CLA up: 332, CLA down: 329, naïve up: 181, naïve down: 252). In AD, more disease-specific ChARs (**Figure 3C**; AD-CLA up: 38, AD-naïve up: 3; AD-CLA down: 68; AD-naïve down: 52) and DEGs (**Figure 4C**; AD-CLA up: 877, AD-naïve up: 448; AD-CLA down: 973; AD-naïve down: 464) were detected. Contrary to AD, Ps had more disease-specific upregulated genes (**Figure 4C**; Ps-CLA up: 157, naïve up: 116; Ps-CLA down: 110, Ps-naïve down: 98). However, more disease-specific downregulated ChAR-s (**Figure 3C**) were found in CD4^+^ naïve (18) compared to CD4^+^CLA^+^ T cells (9), while only a few disease-specific upregulated ChAR-s (**Figure 3C**) were identified in Ps (Ps-CLA: 2; Ps-naïve: 3).

### 3.3 Transcriptome profiling of CD4^+^CLA^+^ T cells from AD and Ps highlights mitotic cell cycle and altered gene expression affecting the JAK–STAT pathway

Next, DEGs of CD4^+^CLA^+^ T cells from AD and Ps were subjected to functional enrichment analysis with STRING (21), focusing on the Reactome (22) database (STR-RCT). First, the top 250 of the most upregulated or downregulated genes from AD and Ps compared to HC were used in analysis, revealing that CD4^+^CLA^+^ T cells from AD show upregulation of numerous genes associated with cell cycle regulation (**Supplementary Figure E4A**). In contrast, upregulated genes from Ps showed the strongest association with immune system-related pathways; however, moderate enrichment of genes from the mitotic cell cycle pathway was also found (**Supplementary Figure E4C**). Surprisingly, in CD4^+^CLA^+^ T cells from AD, genes regulating cytokine signaling, including the IL-4 and IL-13 signaling pathways, were found to be downregulated (**Supplementary Figure E4B**), while downregulated genes from Ps showed functional association with the SMAD2/SMAD3:SMAD4 heterotrimer pathways, suggesting alterations in TGF-β-related signaling (**Supplementary Figure E4D**). In addition, CD4^+^CLA^+^ T cells from both diseases showed downregulation of genes involved in general transcription regulation (**Supplementary Figures E4B, D**).

**Figure 5:**
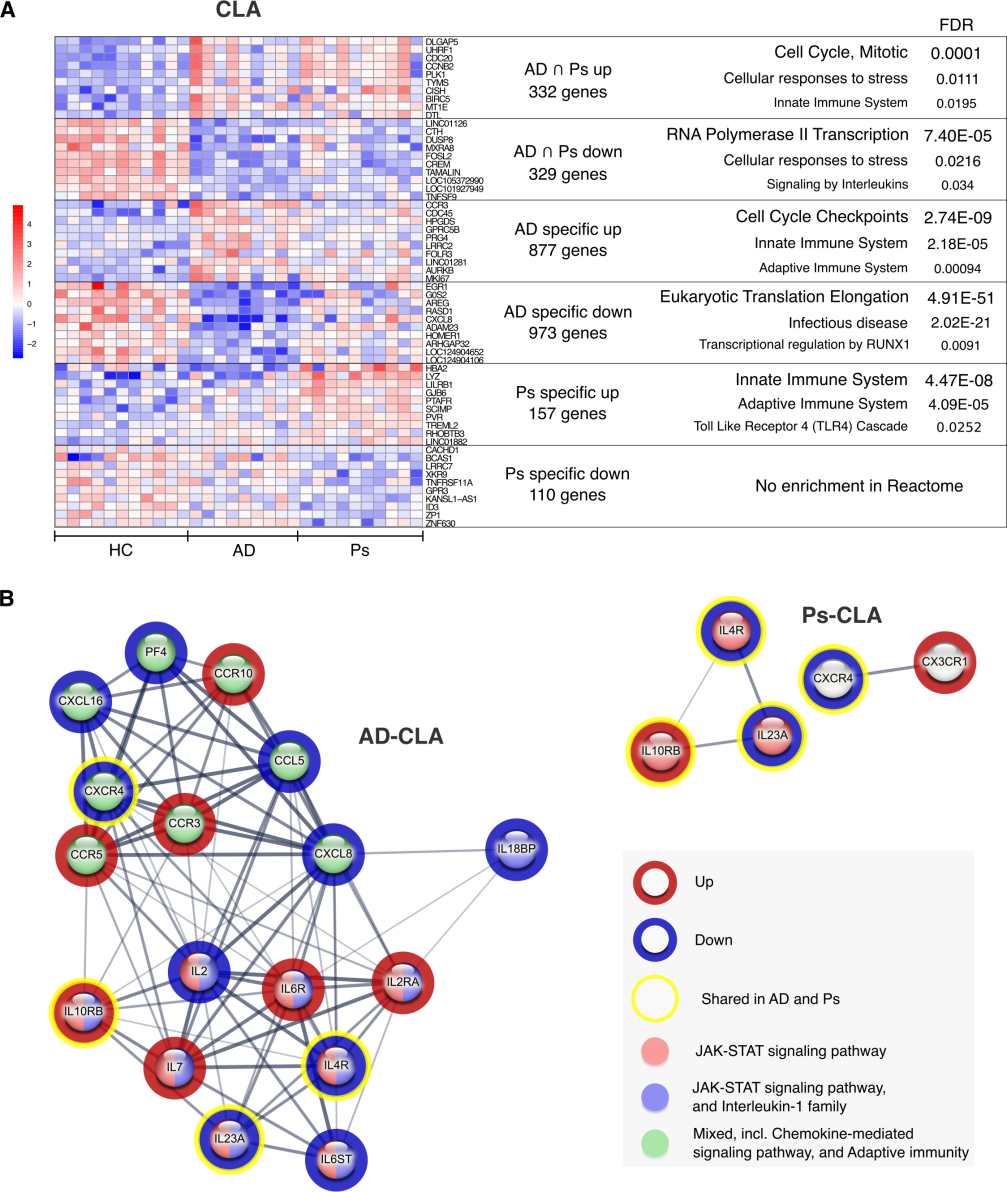
Functional enrichment analysis of DEGs from CD4^+^CLA^+^ T cells (CLA) from patients with AD and Ps. The overlapping and specific DEGs were analyzed for functional enrichment in the Reactome database using the STRING web tool. **(A)** Heatmap (DESeq2 VST-normalized reads, scaled on rows) of the top 10 DEGs from each signature. **(B)** Enrichment analysis of differentially expressed interleukins, chemokines, and related receptors using the local network cluster (STRING) database. Shared (yellow circle) and specific upregulated (red circle) or downregulated (blue circle) genes are presented as a full STRING network (line thickness indicates the strength of data support; minimum required interaction score: medium confidence 0.4). Color-coded nodes indicate corresponding functional groups.

To further look at similarities and differences between AD and Ps, the shared and disease-specific DEGs were subjected to functional enrichment analyses with STR-RCT and Ingenuity Pathway Analysis (IPA). As expected, STR-RCT analysis of shared DEGs of CD4^+^CLA^+^ T cells suggested recent activity of the mitotic cell cycle (**Figure 5A**; **Supplementary Figure E5A**). In line with this, IPA (**Supplementary Figures E8C, D**) highlighted CDKN1A as one of the central regulators supporting the active cell cycle. Among other shared influenced pathways, STR-RCT analysis revealed RNA polymerase II transcription regulation and signaling by interleukins to be associated with the downregulated genes, while cellular response to stress was associated with both up- and downregulated genes (**Figure 5A**, **Supplementary Figure E5**). Although innate immune system-related pathways were associated with shared upregulated genes by STR-RCT analysis (**Figure 5A**; **Supplementary Figure E5**), IPA suggested inhibition of upstream regulators, such as IL1B and TNF (**Supplementary Figures E8C, D**).

STR-RCT analysis of disease-specific DEGs showed many upregulated cell cycle-related genes for AD, including those involved in the regulation of cell cycle checkpoint and active cell cycle (**Figure 5A**; **Supplementary Figure E6A**), while IPA additionally revealed increased cell death (**Supplementary Figure E8A**). In addition, STR-RCT analysis of AD-specific DEGs suggested that immune system-associated pathways are upregulated (**Figure 5A**; **Supplementary Figure E6A**), while pathways related to translation elongation, infectious diseases, and transcriptional regulation by RUNX1 were downregulated (**Figure 5A**; **Supplementary Figure E6B**). Among Ps-specific DEGs, STR-RCT analysis revealed the enrichment of upregulated genes from the innate and adaptive immune system and TLR4 signaling (**Figure 5A**; **Supplementary Figure E7**), while there was no significant enrichment within downregulated genes. In line with this, IPA suggested crosstalk in immune responses regulated by upstream regulators, such as IL1B, TNF, IL6, IL2, and IFNG in Ps (**Supplementary Figure E8B**).

**Figure 6:**
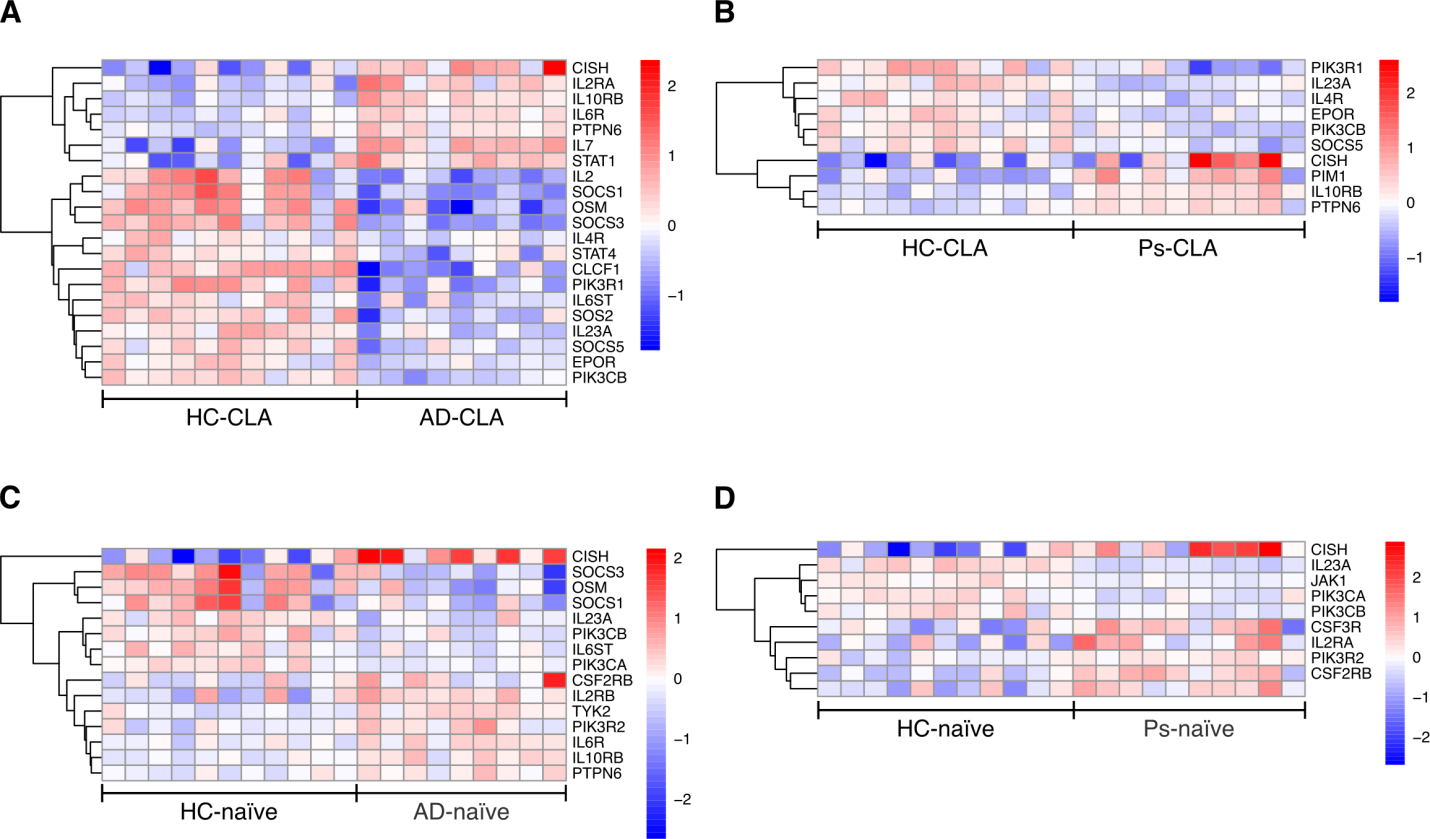
Disease-related alterations in gene expression patterns associated with the JAK–STAT pathway. Heatmaps (with clustering; DESeq2 VST-normalized counts, row-scaled) of differentially expressed genes from the KEGG JAK–STAT signaling pathway (v2026.1.Hs) are shown for CD4^+^CLA^+^**(A, B)** and CD4^+^ naïve **(C, D)** T cells from patients with AD and Ps compared with healthy controls.

Interestingly, among shared DEGs of CD4^+^CLA^+^ T cells from both diseases, several interleukins, chemokines, and related receptors were found, of which *IL23A*, *IL4R*, and *CXCR4* were downregulated and *IL10RB* was upregulated (**Figure 5B**). Of these genes, *IL23A*, *IL4R*, and *IL10RB* were classified as components of the JAK–STAT signaling pathway by the enrichment in the local STRING network cluster database (**Supplementary Figures E12A, B**). In addition, AD-specific genes showed association with interleukin-1 signaling, chemokine signaling, and adaptive immunity (**Figure 5B**; **Supplementary Figure E12A**). Further analysis of DEGs associated with the JAK–STAT pathway revealed disease-related alterations in gene expression, suggesting transcriptomic rewiring of JAK–STAT signaling in CD4^+^CLA^+^ T cells from both diseases (**Figures 6A, B**). Notably, upregulation of *CISH* and downregulation of *SOCS5* were detected in CD4^+^CLA^+^ T cells from patients with AD and Ps. In AD, additional downregulation of *SOCS1*, *SOCS3*, and *STAT4* was observed alongside increased STAT1 expression.

### 3.4 Gene expression signatures of CD4^+^ naïve T cells reveal altered IL-2 and JAK–STAT signaling in AD and Ps

The same strategy as for CD4^+^CLA^+^ T cells was further applied to identify disease-related pathways altered in CD4^+^ naïve T cells from patients with AD and Ps. Interestingly, the top 250 most upregulated genes in AD naïve cells were enriched for immune system-related genes, with a stronger representation of those involved in the regulation to adaptive immunity (**Supplementary Figure E9A**). Minor association of upregulated genes with interferon gamma signaling and MHC II antigen presentation were also identified (**Supplementary Figure E9A**). The top 250 most downregulated genes in AD suggested an association with transcription regulation, with the most significant enrichment for NGF-stimulated transcription (**Supplementary Figure E9B**). In addition, senescence and estrogen signaling were detected as less significant associations (**Supplementary Figure E9B**). The upregulated genes in Ps showed remarkable enrichment for immune system-related genes, especially those regulating innate immunity, of which toll-like receptor signaling can be highlighted (**Supplementary Figure E9C**). Among downregulated genes in Ps, genes involved in transcriptional regulation were significantly enriched (**Supplementary Figure E9D**).

**Figure 7:**
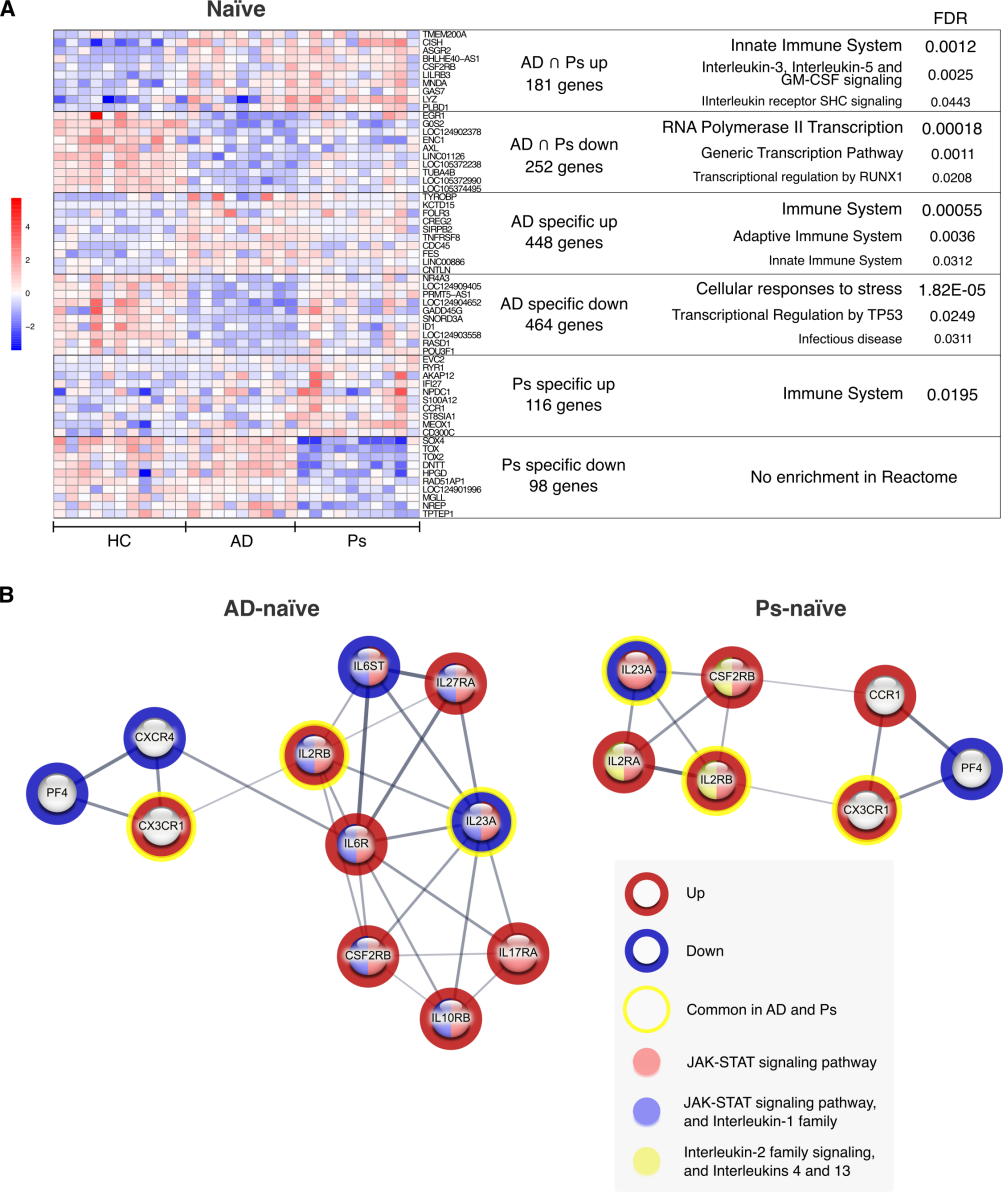
Functional enrichment analysis of transcriptome changes in CD4^+^ naïve T cells (naïve) from patients with AD and Ps. **(A)** The overlapping and specific DEGs were analyzed for functional enrichment in Reactome database using the STRING web tool. Heatmap (DESeq2 VST-normalized reads, scaled on rows) of the top 10 DEGs from each signature. **(B)** Enrichment analysis of differentially expressed interleukins, chemokines, and related receptors using the local network cluster (STRING) database. Shared (yellow circle) and specific genes upregulated (red circle) or downregulated (blue circle) in this category are presented as full STRING network (line thickness indicates the strength of data support; minimum required interaction score: medium confidence 0.4). Color-coded nodes indicate corresponding functional groups.

STR-RCT analysis of shared upregulated genes for AD and Ps revealed enrichment of immune system-related genes, with a stronger association with the innate immune system (**Figure 7A**; **Supplementary Figure E10A**). IPA confirmed the changes in immune system-related pathways and highlighted IL2 as one of the possible upstream regulators of the shared gene expression signature (**Supplementary Figures E11C, D**). STR-RCT analysis of shared downregulated genes predicted influence on general and RUNX1-related transcriptional regulation (**Figure 7A**; **Supplementary Figure E10B**). Among others, IPA suggested ETS1 and KITLG as key shared inhibited upstream regulators, which, in parallel, were suggested to trigger activation through IL-2 and increased cell death via FAS-dependent signaling (**Supplementary Figures E11C, D**). STR-RCT analysis of AD-specific upregulated genes showed enrichment in immune system-related pathways with a leading role in adaptive immunity (**Figure 7A**; **Supplementary Figure E10C**). The AD-specific downregulated genes were associated with transcription regulation and cellular stress (**Figure 7A**; **Supplementary Figure E10D**), with specific but weaker association with transcriptional regulation by TP53 (**Figure 7A**; **Supplementary Figure E10D**). Of note, IPA suggested IL6, TNF, and IL1B among others as potential upstream regulators being inhibited in AD (**Supplementary Figure E11A**). STR-RCT analysis of Ps-specific upregulated genes indicated changes in the immune system, while there was no enrichment found for the downregulated genes (**Figure 7A**; **Supplementary Figure E10E**). Interestingly, IPA suggested the activation of IL33, TNFSF12, IL1A, and IL17A as the top upstream regulators among Ps-specific genes (**Supplementary Figure E11B**).

Analysis of shared DEGs expressing interleukins, chemokines, and related receptors showed *IL23A* being downregulated, while *IL2RB* and *CX3CR1* were upregulated (**Figure 7B**). AD-specific DEGs for interleukins, chemokines, and related receptors were associated with the JAK–STAT signaling pathway and interleukin-1 family as detected by the enrichment in local STRING network cluster database (**Figure 7B**; **Supplementary Figure E12C**). Similar analysis in Ps showed association with JAK–STAT, signaling from interleukin-2 family, and involvement of interleukins 4 and 13 (**Figure 7B**; **Supplementary Figure E12D**). Additional analysis of DEGs associated with the JAK–STAT pathway suggested rewiring of JAK–STAT signaling at the transcriptomic level in CD4^+^ naïve T cells from patients with AD and Ps (**Figures 6C, D**). Similar to CD4^+^CLA^+^ T cells, *CISH* was upregulated in both diseases. Among other findings, downregulation of *SOCS3* and *SOCS1* was observed in CD4^+^ naïve T cells from AD, while *JAK1* was downregulated in CD4^+^ naïve T cells from Ps.

### 3.5 Shared gene expression changes in CD4^+^CLA^+^ and CD4^+^ naïve T cells from patients with AD and Ps indicate systemic inflammation

Since CD4^+^CLA^+^ and CD4^+^ naïve T cells from AD and Ps showed gene expression alterations related to the JAK–STAT signaling pathway, we next focused on DEGs shared in both cell types. We found 300 shared upregulated and 404 shared downregulated genes in AD, while the corresponding numbers in Ps were 89 and 121, respectively. Furthermore, STR-RCT enrichment analysis showed that shared upregulated genes in AD were enriched in signaling pathways related to immune system regulation (**Supplementary Figure E13A**), while downregulated genes were associated with general transcription regulation (**Supplementary Figure E13B**). In Ps, general immune system-related pathways, more specifically, Toll-like receptor signaling was identified (**Supplementary Figure E13C**) for the upregulated genes. Shared downregulated genes in Ps were associated with transcriptional activity of SMAD2/SMAD3:SMAD4 heterotrimer (**Supplementary Figure E13D**). Of note, *CISH*, known as cytokine-inducible SH2-containing protein, was among the top upregulated genes in both diseases and cell types (**Figures 5A**, **7A**), indicating systemic inflammation.

### 3.6 ATAC-seq chromatin accessibility analysis predicts transcription factors that may orchestrate CD4^+^CLA^+^ and CD4^+^ naïve T-cell responses in AD and Ps

**Figure 8:**
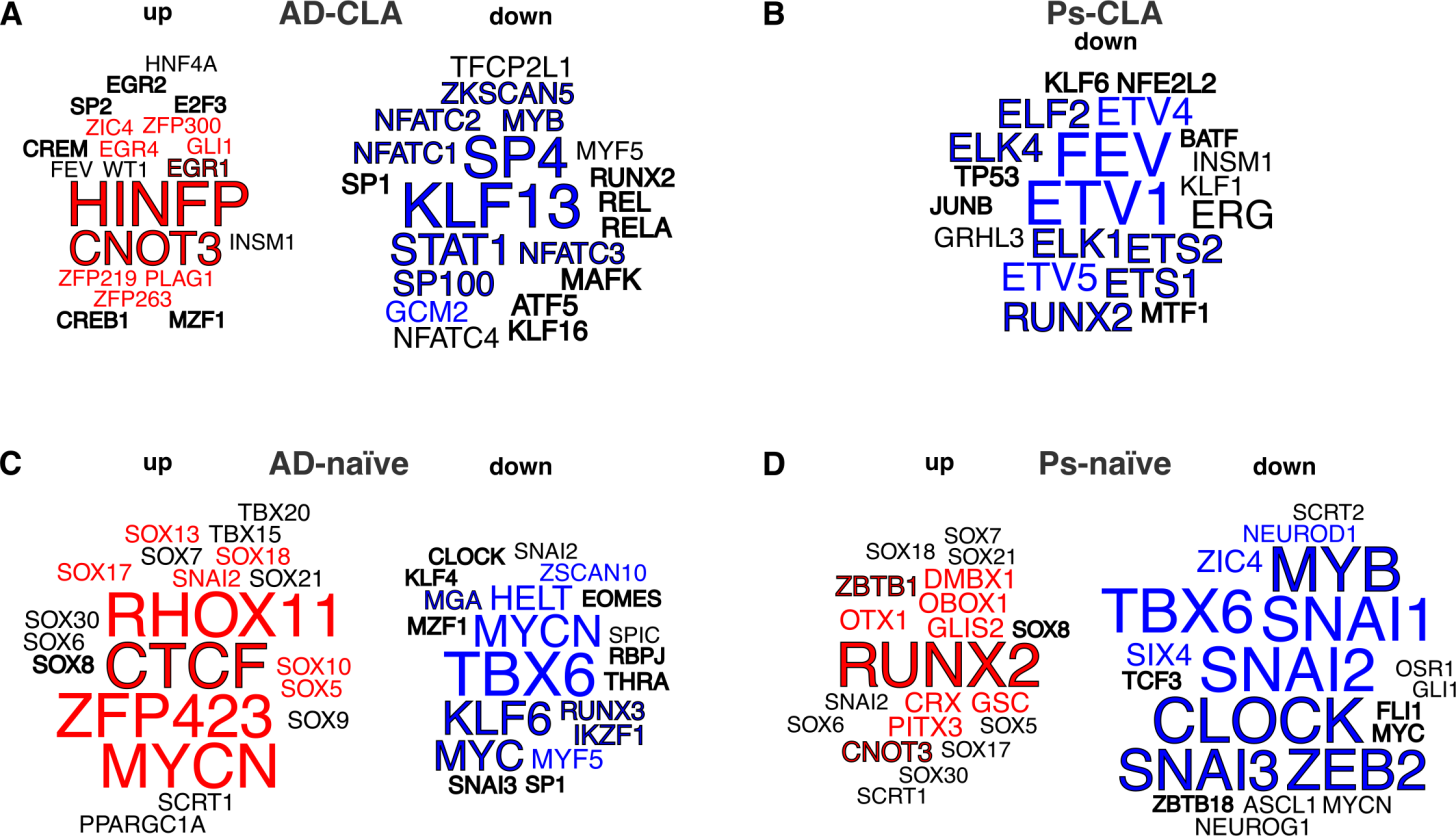
Changes in the accessibility of the chromatin binding sites suggest functionally important transcription factors in CD4^+^CLA^+^ (CLA) and CD4^+^ naïve (naïve) T cells from patients with AD and Ps. **(A–D)** Which TF tool was used to identify TFs using more open (up) or more closed (down) chromatin regions from ATAC-seq in AD and Ps. No association was found with Ps CLA up regions. The top 20 TFs with the highest rank are presented as word clouds, where the top 10 up (red) or down (blue) regions are highlighted. TFs with confirmed expression by transcriptomic analysis are marked with dark edges.

Given the limited number of differential ChARs, integrated analysis of RNA-seq and ATAC-seq showed overlap between DEGs and ChARs in relatively few annotated genes (**Supplementary Table E7**). Therefore, we next focused on TF binding sites and analyzed differential ChARs identified in AD and Ps using the WhichTF (20) tool, which applies an ontology-guided functional approach to find context-specific TFs. We focused on the top 20 TFs with the highest ranks and considered those with confirmed expression based on RNA-seq and ranked among the top 10 as the most biologically relevant (**Figure 8**). However, as epigenetic landscape may be associated with the past or future events in gene expression regulation, all top 20 ranked TFs were included in the functional enrichment analysis using the local STRING network cluster database.

Interestingly, the binding sites for the top 20 TFs suggested active regulation in association with early growth response for AD CLA^+^ cells (**Figure 8A**; **Supplementary Figure E14A**), whereas negative regulation of calcium ion import across the plasma membrane was linked to the more closed chromatin areas (**Figure 8A**; **Supplementary Figure E14B**). Consistent with this, analysis of TFs with confirmed expression from both more open and closed regions highlighted the same pathways, among others (**Supplementary Figure E15A**). Similar analysis for Ps revealed TFs only for the more downregulated regions (**Figure 8B**), while functional association analysis suggested a connection between these TFs and granulocyte differentiation (**Supplementary Figures E14C**, **E15B**).

In addition, altered ChAR in naïve cells from AD and Ps suggested overlap with mesenchyme development with regard to the TF involved (**Figures 8C, D**, **Supplementary Figures E14D–F**), while similar analysis for TFs with confirmed expression proposed influence on general transcription regulation in both diseases (**Figure 8C, D**; **Supplementary Figure E15C, D**) and the association with mesenchyme development for AD (**Figure 8C**; **Supplementary Figure E15C**).

## 4 Discussion

Although the pivotal role of CD4^+^CLA^+^ T cells in AD and Ps is well documented (8, 9), the studies that compare changes in CD4^+^CLA^+^ T cells between the two diseases or with disease-associated alterations in other T-cell subsets are very limited. Here, we show that both the CD4^+^CLA^+^ and CD4^+^ naïve T cells display transcriptomic and epigenetic alterations in AD and Ps, with partially overlapping as well as disease-specific pathways being affected.

In a comparative analysis of the two diseases, we first focused on differences in the proportions of cell types. We observed a higher proportion of CD4^+^ naïve T cells among all CD4^+^ T cells and confirmed the increased frequency of Treg cells (14) among the CD4^+^CLA^+^ T-cell subsets in AD. Although we did not detect an increased proportion of CD4^+^CLA^+^ T cells for the studied diseases (23), we observed a higher proportion of CCR7-expressing cells among CD4^+^CLA^+^ T cells from patients with Ps, consistent with previous findings (24), suggesting a potential involvement of central memory-like features in Ps. Further analysis of the CD4^+^CLA^+^ T-cell population revealed increased CCR7 expression levels specifically on CD4^+^CLA^+^ Tregs from patients with Ps. Although CCR7 is commonly associated with T_CM_ cells, these changes appear to be confined to the Treg subset rather than reflecting alterations in the broader central memory compartment. As CCR7 mediates migration to secondary lymphoid organs, this finding suggests altered migratory properties of skin-homing Treg cells and supports previous reports of Treg dysfunction in Ps (25, 26). Considering that Treg cells in Ps may preferentially recirculate through lymphoid organs rather than migrating into peripheral tissues, these findings collectively suggest reduced Treg activity at sites of inflammation and impaired suppression of skin inflammation. In contrast, in AD, the mechanisms driving skin inflammation appear to differ, potentially involving active but insufficient regulatory responses rather than altered migratory behavior. Consistent with previous reports that circulating CD4^+^CD25^+^ Tregs are expanded in AD (14, 27), we found that CLA^+^ skin-homing and naïve Treg subsets exhibit higher CD25 expression levels. Overall, our results are consistent with previous findings that patients with Ps exhibit skewed T-cell distributions, including increased circulating CD4^+^CCR7^+^ T cells that may contribute to disease persistence (24), whereas in AD, Treg frequency reflects immune dysregulation and has been positively correlated with disease severity (14).

As expected, we observed that transcriptomic and epigenetic landscapes differ markedly between CD4^+^CLA^+^ and CD4^+^ naïve T cells in all study groups. However, disease-associated transcriptomic changes were more pronounced within these T-cell populations compared with epigenetic alterations in both AD and Ps. When we subjected DEGs from CD4^+^CLA^+^ and CD4^+^ naïve T cells to pathway analyses, we observed involvement of JAK–STAT signaling in both diseases and cell types. Interestingly, although activation of the JAK–STAT pathway is a well-known feature in AD and Ps (28, 29), our results do not directly point to the classical activation of JAK–STAT but rather suggest a transcriptomic rewiring of this signaling in cytokine-driven chronic inflammation. Among other highlights, CD4^+^CLA^+^ cells had gene expression changes linked to mitotic cell cycle, especially in AD, while a stronger link with innate immune activation was found in Ps than in AD. Notably, for CD4^+^ naïve T cells, transcriptomic changes were associated with IL-2 signaling in both diseases. Flow cytometric analysis of CD25 (IL2RA) expression revealed increased levels in AD in both CD4^+^CLA^+^ skin-homing and CD4^+^ naïve Treg subsets, whereas no changes in CD25 expression were observed in Ps. At the same time, there were no differences in the frequencies of CD4^+^ naïve Treg cells between the diseases and HC. Together, these findings suggest increased sensitivity for IL-2 due to increased receptor expression in AD affecting both CD4^+^CLA^+^ and CD4^+^ naïve T cells and highlight the disease-associated changes in Treg subsets. In contrast, CD4^+^ naïve T cells in Ps display a transcriptionally altered profile indicative of a primed state for IL-2 responsiveness, without the corresponding increase of CD25 at the protein level.

When we focused on CD4^+^CLA^+^ T cells, we observed that *CXCR4*, a marker associated with migration to the lymph nodes (30), was downregulated in both diseases, while chemokine receptors responsible for T-cell migration to inflammatory sites [*CCR5* (31) associated primarily with the Th1 phenotype and *CCR3* (31, 32) associated with the Th2 phenotype] and to the skin [*CCR10* (33, 34)] were increased in AD and [*CX3CR1* (35, 36)] in Ps. This, together with enrichment of pathways related to mitotic activity, indicates that CD4^+^CLA^+^ T cells from AD and Ps may be shifted from T_CM_ to T_EM_. In line with this, upregulation of *IL6R* (37) and *IL2RA* (38) in AD, known factors contributing to survival and expansion of T cells, also points to the disease-related activation state of CD4^+^CLA^+^ T cells. Interestingly, within the shared DEGs, *IL4R* was downregulated, while *IL10RB* was upregulated, additionally suggesting that CD4^+^CLA^+^ T cells from both diseases attempt to cope with increased systemic inflammation by rewiring gene expression in the JAK–STAT pathway at the transcriptomic level.

Although our main focus was CD4^+^CLA^+^ T cells, it is noteworthy that we observed significant gene expression changes also in CD4^+^ naïve T cells from AD and Ps. To our best knowledge, this finding is novel for AD and Ps; however, it is consistent with recent studies showing that, in particular cases, such as autoimmunity, CD4^+^ naïve T cells may harbor poised and skewed subpopulations (39–41) Our results suggest primed state for IL-2/STAT5 responsiveness in CD4^+^ naïve T cells in Ps, whereas in AD, the findings are consistent with enhanced IL-2 signaling. This priming likely lowers the threshold for these cells to differentiate into effector or regulatory lineage (42–44), while simultaneous upregulation of *CX3CR1* in diseased conditions in CD4^+^ naïve cells suggest shift toward the effector phenotype (35). As IL-2 is known to signal through the JAK1/JAK3-STAT5 axis, involvement of the JAK–STAT pathway may be linked to IL-2 signaling, which is pivotal in regulating T-cell proliferation, differentiation, and survival (38, 45).

Interestingly, we detected decreased expression of *IL23A* in both diseases and analyzed cell types, which could be a result of the influence of the inflammatory environment in AD and Ps, as T cells are not the primary source for IL-23 (46). Another intriguing finding was the disease-related increase in *CISH* expression, which ranked among the top upregulated genes in both CD4^+^CLA^+^ and CD4^+^ naïve T cells in AD and Ps. Although *CISH* is best known as a member of the suppressor of cytokine signaling family proteins, it has also been linked to accelerated immune aging and suggested as a potential target to reduce inflammaging (47). This coincides well with the increased low-level chronic inflammation characteristics of AD and Ps, potentially contributing to immune system dysregulation.

In line with transcriptome analyses, the ATAC-seq TF binding site analysis from both analyzed cell types and conditions indicated dysfunctional regulation under chronic inflammatory conditions, while there was no obvious association with T-cell proliferation or differentiation, suggesting that those pathways may be regulated at the transcriptional or post-transcriptional level. Among the relatively few overlaps we detected between the transcriptome and ATAC-seq results, we found that the TF IKZF1 was associated with the more closed regions in CD4^+^ naïve T cells in AD, while its expression was decreased in CD4^+^ naïve T cells in both diseases. IKZF1 is needed for normal differentiation and activation of T cells, and lack of it could promote inflammatory phenotypes in CD4^+^ naïve T cells in diseased conditions (48–50). In CD4^+^CLA^+^ T cells in AD, increased *HINFP* expression was detected coherently with its association with more open chromatin areas. HINFP is linked to increased mitotic activity (51, 52), which aligns well with the transcriptional profile enriched for cell cycle-associated DEGs in CD4^+^CLA^+^ cells from AD in the current study. Interestingly, STAT1 binding sites showed associations with more closed chromatin regions in CD4^+^CLA^+^ T cells in AD, which is in line with Th2 skewing and previous knowledge on the role of JAK–STAT signaling in AD (53). This finding may also indicate changes in CD4^+^ T-cell effector or memory functions (54, 55). A simultaneous increase in *STAT1* mRNA expression in CD4^+^CLA^+^ T cells from AD may be associated with the rewiring of JAK–STAT signaling at the transcriptomic level. In CD4^+^CLA^+^ T cells from Ps, ETS1, which is involved in Treg functions (56), was associated with the more closed regions. In CD4^+^ naïve T cells in Ps, RUNX2, a protein not directly linked to naïve T-cell status (57), while important in T-cell development in thymus (58), was found to be associated with open regions.

Our study also has several limitations. First, the heterogeneity of the CD4^+^CLA^+^ T-cell population containing T_CM_, T_EM_, and Tregs may have an influence on our results. It should also be noted that the patient cohort used included only male patients and that the age of recruited individuals was variable. Moreover, although patients were treatment-free for at least 2 weeks prior to recruitment, we cannot exclude the possibility that the use of recent medications may have influenced the transcriptomic and epigenetic changes we observed. In addition, the potential significance of highlighted TFs should be further validated for their functional effects, while functional studies are also needed to determine whether the shared upregulated genes in CD4^+^ T-cell subsets, including CISH, may have potential as drug targets. Similarly, the transcriptomic findings suggesting systemic inflammation, the rewiring of JAK–STAT signaling, and the recent mitotic activity of CD4^+^CLA^+^ T cells should be validated at the protein level to fully confirm these observations.

In summary, our findings reveal distinct transcriptomic and epigenetic landscapes of CD4^+^CLA^+^ and naïve T cells in AD and Ps, indicating transcriptomic rewiring of the JAK–STAT pathway in both cell types. The study highlights increased IL-2 sensitivity in CD4^+^ naïve T cells from AD and a transcriptionally primed state for IL-2 responsiveness in Ps, all indicative of ongoing chronic inflammation.

## Data Availability

The datasets presented in this study can be found in online repositories. The names of the repository/repositories and accession number(s) can be found in the article/**Supplementary Material**.
