# Supplementary material for: Transcriptomic rewiring of the JAK–STAT pathway in circulating CD4+CLA+ and CD4+ naïve T cells from patients with atopic dermatitis and psoriasis
Source: Front Immunol. 2026 Apr 20;17:1782684. doi: 10.3389/fimmu.2026.1782684 (PMC13136270; doi:10.3389/fimmu.2026.1782684)
Supplement: Supplementary file 1 [file DataSheet1.pdf]

Supplementary Material

1 Supplementary Tables and Figures

1.1 Supplementary Tables

Table E1. Descriptive characteristics of the patients and healthy controls

	AD (ATAC-seq)	AD (RNA-seq)	Ps	HC
Sampe size (n)[1]	10	9	10	11
Age (years)[2]	26 [19.5-41.25]	25.5 [19-43]	53.5 [45.25-56.75]	44 [39-44.5]
Disease severity (mLEASI)[2]	22.8 [21.6-27.375]	22.5 [10.8-22.8]	NA	NA
Disease severity (PASI)[2]	NA	NA	17.6 [12.3-23.1]	NA

AD: atopic dermatitis
Ps: psoriasis vulgaris
HC: healthy control
mLEASI: modified Local Eczema and Severity Index
PASI: Psoriasis Area and Severity Index
[1] Only males were included in the study
[2] Data are presented as median [0.25-0.75 quartiles]

Table E2. Patients and healthy controls from BIOMAP ADPSHC cohort used in the current study.

BIOMAP SUBJECT ID	AGE	SEX	Dx	ATAC-seq	RNA-seq
BIOMAP_ADPSCN_UTARTU_10000011	26	M	AD	+	-
BIOMAP_ADPSCN_UTARTU_10000012	30	M	AD	+	+
BIOMAP_ADPSCN_UTARTU_10000013	47	M	AD	+	-
BIOMAP_ADPSCN_UTARTU_10000015	19	M	AD	+	+
BIOMAP_ADPSCN_UTARTU_10000016	21	M	AD	+	+
BIOMAP_ADPSCN_UTARTU_10000018	43	M	AD	+	+
BIOMAP_ADPSCN_UTARTU_10000019	36	M	AD	+	+
BIOMAP_ADPSCN_UTARTU_10000020	18	M	AD	+	+
BIOMAP_ADPSCN_UTARTU_10000005	43	M	AD	+	+
BIOMAP_ADPSCN_UTARTU_10000009	18	M	AD	+	+
BIOMAP_ADPSCN_UTARTU_10000007	58	M	AD	-	+
BIOMAP_ADPSCN_UTARTU_20000011	49	M	Ps	+	+
BIOMAP_ADPSCN_UTARTU_20000012	62	M	Ps	+	+
BIOMAP_ADPSCN_UTARTU_20000013	56	M	Ps	+	+
BIOMAP_ADPSCN_UTARTU_20000014	58	M	Ps	+	+
BIOMAP_ADPSCN_UTARTU_20000018	25	M	Ps	+	+
BIOMAP_ADPSCN_UTARTU_20000003	37	M	Ps	+	+
BIOMAP_ADPSCN_UTARTU_20000004	57	M	Ps	+	+
BIOMAP_ADPSCN_UTARTU_20000007	55	M	Ps	+	+
BIOMAP_ADPSCN_UTARTU_20000008	44	M	Ps	+	+
BIOMAP_ADPSCN_UTARTU_20000009	52	M	Ps	+	+
BIOMAP_ADPSCN_UTARTU_30000614	26	M	HC	+	+
BIOMAP_ADPSCN_UTARTU_30000630	42	M	HC	+	+
BIOMAP_ADPSCN_UTARTU_30000631	45	M	HC	+	+

BIOMAP_ADPSCN_UTARTU_30000655	44	M	HC	+	+
BIOMAP_ADPSCN_UTARTU_30000658	59	M	HC	+	+
BIOMAP_ADPSCN_UTARTU_30000666	44	M	HC	+	+
BIOMAP_ADPSCN_UTARTU_30000683	19	M	HC	+	+
BIOMAP_ADPSCN_UTARTU_30000690	41	M	HC	+	+
BIOMAP_ADPSCN_UTARTU_30000703	37	M	HC	+	+
BIOMAP_ADPSCN_UTARTU_30000741	49	M	HC	+	+
BIOMAP_ADPSCN_UTARTU_30000764	44	M	HC	+	+

Dx: Diagnosis/Disease
AD: atopic dermatitis
Ps: psoriasis vulgaris
HC: healthy control
mLEASI: modified Local Eczema and Severity Index
PASI: Psoriasis Area and Severity Index

Table E3. Disease and treatment characteristics of patients from BIOMAP ADPSHC cohort used in the current study.

**BIOMAP SUBJECT ID**	**mLEASI**	**PASI**	**Dx**	**Dx DURATION (years)**	**TREATMENT (most recent)**
BIOMAP_ADPSCN_UTARTU_10000011	32.8	NA	AD	23	Elocon, Protopic, Cyclosporine
BIOMAP_ADPSCN_UTARTU_10000012	32.2	NA	AD	29	Advantan, Cyclosporine
BIOMAP_ADPSCN_UTARTU_10000013	27.5	NA	AD	46	Elocon, Cyclosporine
BIOMAP_ADPSCN_UTARTU_10000015	27	NA	AD	19	Prednisolone
BIOMAP_ADPSCN_UTARTU_10000016	8.8	NA	AD	20	*topical corticosteroids and calcineurin inhibitors*
BIOMAP_ADPSCN_UTARTU_10000018	22.8	NA	AD	30	Cyclosporine
BIOMAP_ADPSCN_UTARTU_10000019	21.3	NA	AD	35	Tacrolimus
BIOMAP_ADPSCN_UTARTU_10000020	6.4	NA	AD	16	Advantan, *antihistamines*
BIOMAP_ADPSCN_UTARTU_10000005	22.8	NA	AD	1	Prednisolone
BIOMAP_ADPSCN_UTARTU_10000009	22.5	NA	AD	7	Cyclosporine
BIOMAP_ADPSCN_UTARTU_10000007	10.8	NA	AD	6	Prednisolone
BIOMAP_ADPSCN_UTARTU_20000011	NA	11.2	Ps	31	Diprosalic
BIOMAP_ADPSCN_UTARTU_20000012	NA	11.8	Ps	27	Trexan
BIOMAP_ADPSCN_UTARTU_20000013	NA	13.8	Ps	9	Elocon, Daivobet, Sulf-Salic
BIOMAP_ADPSCN_UTARTU_20000014	NA	26.8	Ps	26	Cyclosporine
BIOMAP_ADPSCN_UTARTU_20000018	NA	24	Ps	8	Elocon, Daivobet
BIOMAP_ADPSCN_UTARTU_20000003	NA	26.8	Ps	20	Daivobet, Dermovate
BIOMAP_ADPSCN_UTARTU_20000004	NA	20.4	Ps	19	Daivobet
BIOMAP_ADPSCN_UTARTU_20000007	NA	17.6	Ps	33	Daivobet

BIOMAP_ADPSCN_UTARTU_20000008	NA	17.6	Ps	9	Daivobet
BIOMAP_ADPSCN_UTARTU_20000009	NA	9.5	Ps	23	Daivobet, Diprosalic

Dx: Diagnosis/Disease
AD: atopic dermatitis
Ps: psoriasis vulgaris
HC: healthy control
mLEASI: modified Local Eczema and Severity Index
PASI: Psoriasis Area and Severity Index

Table E4. Antibodies in the antibody staining mix for cell sorting.

Marker	Fluorochrome	Clone	Antibody	Company	Final dilution
CLA	FITC	HECA-452	321396	BioLegend	1 : 50
CD4	AF700	OKT4	317426	BioLegend	3 : 1000
CCR7	PE-Dazzle	G043H7	353236	BioLegend	1 : 50
CD45RA	PE-Cy7	HI100	304125	BioLegend	1 : 500
CD8	BV605	RPA-T8	301040	BioLegend	1.5 : 100
CD3	APC	OKT3	317318	BioLegend	1.5 : 100
CD25	PE	BC96	302606	BioLegend	1 : 20
CD127	BV421	A019D5	351310	BioLegend	1 : 50

Table E5. Cell sorter (LE-MA900FP, Sony) measurement settings

Laser	Channel	Sensor gain	Marker	Fluorochrome
488	FSC	5	-	-
488	BSC	33.80%	-	-
488	FL1	42.00%	CLA	FITC
488	FL2	41.00%	CD25	PE
488	FL3	46.50%	CCR7	PE-Dazzle
488	FL4	40.00%	Live/Dead	7-AAD
488	FL5	48.50%	CD45RA	PE-Cy7
405	FL6	40.00%	CD127	Brilliant Violet 421
405	FL9	48.00%	CD8	Brilliant Violet 605
638	FL10	46.00%	CD3	APC
638	FL11	46.50%	CD4	Alexa Fluor 700

Table E6. Cell sorter spillover matrix (%) for compensation setup

	CLA: FITC	CD25: PE	CCR7: PE-Dazzle	Live/ Dead: 7-AAD	CD45RA: PE-Cy7	CD127: Brilliant Violet 421	CD8: Brilliant Violet 605	CD3: APC	CD4: Alexa Fluor 700
CLA:FITC	100	8.62	5.12	0.2	0.1	0	0.09	-0.01	-0.03
CD25:PE	2.04	100	54.87	2.59	1.4	-0.01	2	-0.06	-0.17
CCR7:PE-Dazzle	0.3	16.48	100	7.4	3.93	0	3.24	0.36	0.29

Live/Dead:7-AAD	0	0	0	100	0	0	0	0	0
CD45RA:PE-Cy7	0.14	0.97	0.41	0.08	100	0.02	-0.08	-0.08	0.24
CD127:Brilliant Violet 421	-0.03	0.01	-0.16	-0.02	-0.03	100	0.36	-0.04	-0.07
CD8:Brilliant Violet 605	-0.09	22.25	73.06	6.88	5.01	2.83	100	19.75	16.63
CD3:APC	-0.02	0.02	0.48	6.89	4.75	0	2.39	100	61.63
CD4:Alexa Fluor 700	0.12	0.03	0.01	2.59	6.24	0.03	-0.04	0.77	100

Table E7. Overlap of annotated genes between differential ChARs from ATAC-seq and DEGs from RNA-seq. ATAC-seq narrow peaks were annotated using GREAT (single nearest gene: 1000000 bp max extension, curated regulatory domains included), peak distances from transcription start site (TSS) are shown accordingly.

Condition	Gene ID	Peak distance from gene TSS (bp)	ATAC-seq: log2(FC)	ATAC-seq: P-adj	RNA-seq: log2(FC)	RNA-seq: P-adj
AD-CLA	FOSL2	+17107	-0.5	3.29E-02	-2.23	1.03E-09
AD-CLA	ZFP36L2	+92268	-0.43	3.63E-02	-1.25	1.27E-09
AD-CLA	ZFP36L2	+67623	-0.38	4.34E-02	-1.25	1.27E-09
AD-CLA	BACH2	+62624	-0.81	6.81E-03	-1.07	2.17E-07
AD-CLA	RIPK2	-28169	-0.62	4.34E-02	-1.31	7.83E-07
AD-CLA	PITPNC1	+56316	-0.44	4.72E-02	-0.68	3.42E-06
AD-CLA	CXCR4	-122665	-0.67	4.48E-02	-1.08	8.29E-06
AD-CLA	PWWP2B	+50316	-0.68	1.83E-05	-1.18	1.71E-05
AD-CLA	CCDC141	+392	0.59	3.84E-02	1.1	5.62E-05
AD-CLA	JUND	-8940	-0.59	3.91E-02	-0.99	1.04E-04
AD-CLA	TSPAN18	-83219	-0.66	2.65E-02	-0.8	2.94E-04
AD-CLA	SUSD4	-493	-0.45	1.56E-02	-1.45	4.57E-04
AD-CLA	OSM	-424	-0.66	1.79E-02	-1.3	4.83E-04
AD-CLA	TM9SF2	-84449	-0.93	2.56E-02	0.53	1.14E-03
AD-CLA	TM9SF2	-66858	-0.51	2.95E-02	0.53	1.14E-03
AD-CLA	SOX8	+15897	-0.67	3.79E-02	-1.22	1.55E-03
AD-CLA	B4GALT1	+7961	-0.7	4.85E-06	-0.68	2.22E-03
AD-CLA	RPS16	+4962	-0.56	4.33E-02	-0.54	3.11E-03
AD-CLA	FKBP11	+587	-0.53	1.16E-03	-0.43	3.35E-03
AD-CLA	RMDN1	+36159	0.68	2.33E-02	0.34	3.60E-03
AD-CLA	USP46	+70421	-0.81	6.81E-03	-0.98	4.18E-03
AD-CLA	BLM	-30700	0.77	3.90E-02	0.71	6.08E-03
AD-CLA	CFAP20	+2229	0.67	1.05E-02	0.42	7.86E-03
AD-CLA	BHLHE40	-157000	-0.54	2.17E-02	-0.99	8.35E-03
AD-CLA	BHLHE40	-110387	-0.41	3.19E-02	-0.99	8.35E-03
AD-CLA	BHLHE40	+5059	-0.77	3.94E-02	-0.99	8.35E-03
AD-CLA	SLC1A1	+40485	0.63	1.42E-02	1.3	1.03E-02
AD-CLA	BNIP2	-125153	-0.97	8.11E-04	-0.45	1.06E-02
AD-CLA	PBX4	+12737	-0.72	2.56E-02	-0.5	1.11E-02

AD-CLA	SLC27A3	+30254	-0.99	9.51E-03	0.72	1.44E-02
AD-CLA	NR1H3	-13661	-0.97	5.68E-04	0.67	1.53E-02
AD-CLA	SORL1	+12435	-0.73	1.22E-02	-0.26	2.32E-02
AD-CLA	SORL1	+16001	-0.71	3.29E-02	-0.26	2.32E-02
AD-CLA	STX3	+12087	-0.81	4.22E-02	-0.62	2.55E-02
AD-CLA	NLRP3	-10502	-0.68	9.93E-03	-0.96	2.84E-02
AD-CLA	PANK1	+30120	-0.81	6.81E-03	-0.82	4.95E-02
Ps-CLA	OAF	+362	0.71	2.45E-05	1.4	7.94E-06
Ps-CLA	B4GALT1	+7961	-0.53	4.96E-06	-0.79	6.93E-04
Ps-CLA	SPTBN1	+116489	-0.79	8.78E-06	-0.46	2.96E-03
AD-Naïve	JUNB	-1411	-0.65	5.58E-03	-1.7	2.55E-11
AD-Naïve	SKI	+2860	-0.75	4.76E-03	-1.24	6.88E-07
AD-Naïve	SKI	+3414	-0.78	1.01E-03	-1.24	6.88E-07
AD-Naïve	SKI	+72244	-0.4	1.18E-02	-1.24	6.88E-07
AD-Naïve	TNFRSF1B	+6764	-0.46	1.52E-02	0.76	9.56E-07
AD-Naïve	DUSP2	-134	-0.64	3.82E-03	-1.26	3.97E-06
AD-Naïve	FOS	+4442	-1.2	1.52E-02	-1.88	1.05E-03
AD-Naïve	LEPROTL1	+4812	-0.48	3.52E-02	-0.59	1.53E-03
AD-Naïve	FOSL2	-2332	-0.56	4.97E-02	-1.31	2.55E-03
AD-Naïve	CD44	+352	-0.36	4.39E-02	-0.26	1.09E-02
AD-Naïve	WNT7A	+13420	-0.55	6.03E-03	-0.79	1.27E-02
AD-Naïve	PTGER2	+37884	0.96	1.00E-02	1.1	1.29E-02
AD-Naïve	CSRNP1	+619	-0.35	3.77E-02	-0.76	1.44E-02
AD-Naïve	BTG2	-15342	-0.34	4.67E-02	-0.58	1.50E-02
AD-Naïve	ITPKB	+75890	-0.55	1.26E-02	-0.5	1.86E-02
AD-Naïve	ITPKB	+48174	-0.7	2.65E-02	-0.5	1.86E-02
AD-Naïve	CXCR4	-680	-0.48	1.04E-03	-0.63	2.47E-02
AD-Naïve	CXCR4	-2622	-0.35	2.08E-02	-0.63	2.47E-02
AD-Naïve	CXCR4	-266260	-0.65	2.28E-02	-0.63	2.47E-02
AD-Naïve	GPR132	-28133	-0.5	4.61E-02	-0.63	3.28E-02
AD-Naïve	RBM38	+1504	-0.5	1.26E-02	-0.51	3.30E-02
AD-Naïve	ARRDC2	-6930	-0.51	5.58E-03	-0.47	3.66E-02
AD-Naïve	ARRDC2	+15750	-0.64	9.85E-03	-0.47	3.66E-02
Ps-Naïve	LEPROTL1	-277	-0.68	7.26E-03	-0.59	1.98E-03
Ps-Naïve	ARRDC2	+15750	-0.72	7.08E-03	-0.6	6.79E-03

1.2 Supplementary Figures

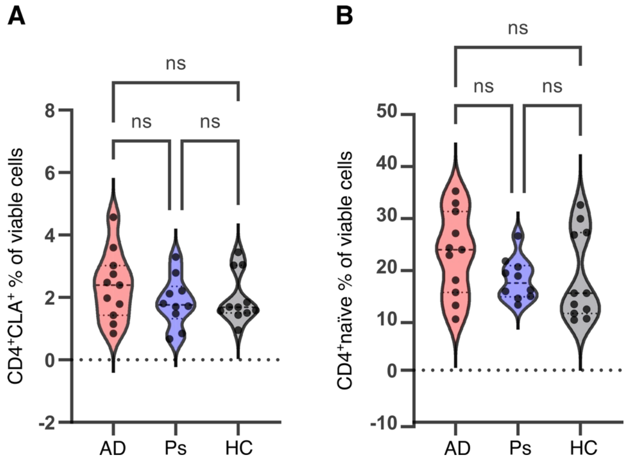

Figure E1. Proportions of $CD4^{+}CLA^{+}$ or $CD4^{+}$ naïve T cells from PBMC-s are not changed in AD or Ps. Proportions (%) as measured before cell sorting from live cells by flow cytometry. Proportions of $CD4^{+}CLA^{+}$ T cells (A) or $CD4^{+}$ naïve T cells (B) from live cells in AD, Ps and HC.

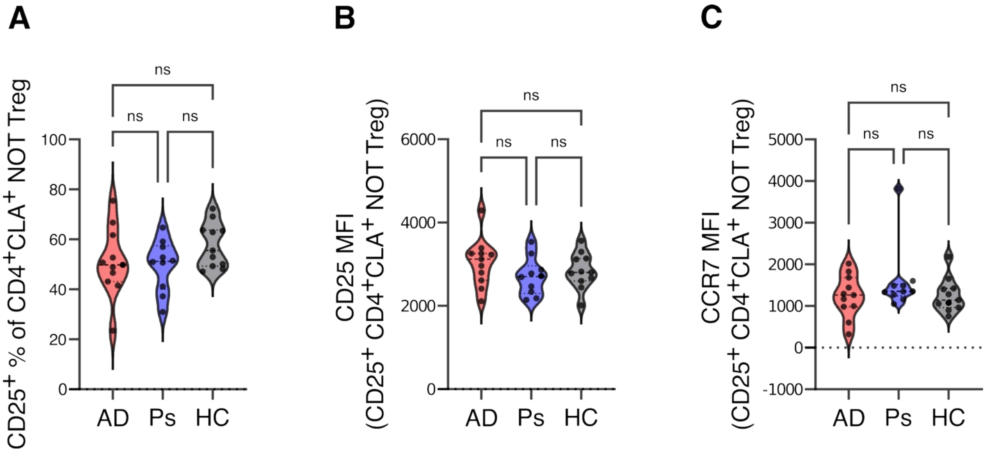

Figure E2. $CD25^{+}$ cells from $CD4^{+}CLA^{+}$ T cells without Tregs do not show differences in CD25 expression between AD, Ps or HC. Proportion (%) as measured before cell sorting from live cells by flow cytometry. Proportions of $CD25^{+}$ T cells from $CD4^{+}CLA^{+}$ T cells without Tregs (**A**). Median fluorescence itensity (MFI) of CD25 (**B**) or CCR7 (**C**) from $CD4^{+}CLA^{+}$ T cells without Tregs in AD, Ps and HC.

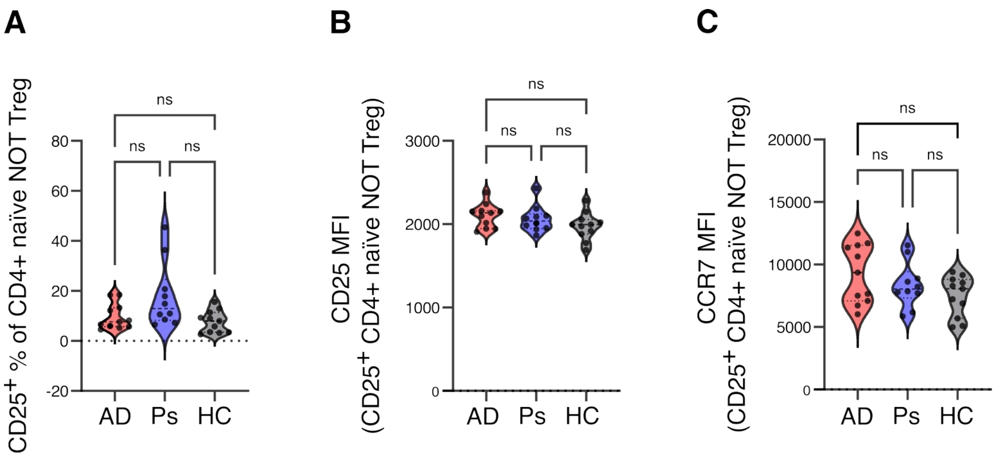

Figure E3. $CD25^+$ cells from $CD4^+$ naïve T cells without naïve Tregs do not show differences in CD25 expression between AD, Ps or HC. Proportion (%) as measured before cell sorting from live cells by flow cytometry. Proportions of $CD25^+$ cells from $CD4^+$ naïve T cells without naïve Tregs (**A**). Median fluorescence itensity (MFI) of CD25 (**B**) or CCR7 (**C**) from $CD4^+$ naïve T cells without naïve Tregs in AD, Ps and HC.

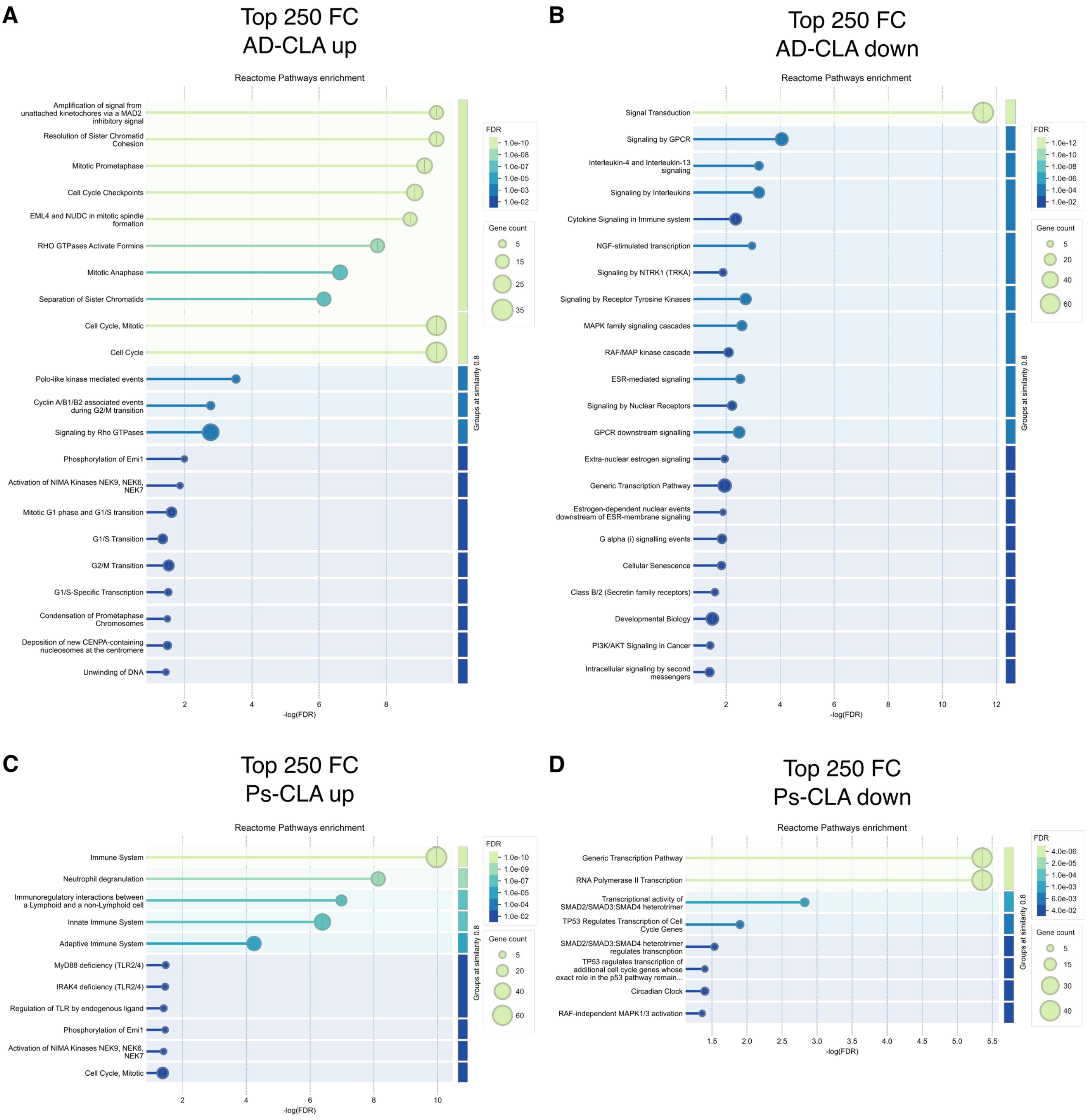

Figure E4. DEGs of CD4^{+}CLA^{+} T cells (CLA) from AD and Ps are associated with immune sytem and cell cycle associated pathways. Pathway enrichment analysis was performed with STRING web tool using Reactome database with top 250 most up- or donwregulated genes in AD-CLA (**A** and **B**) and Ps-CLA (**C** and **D**) cells.

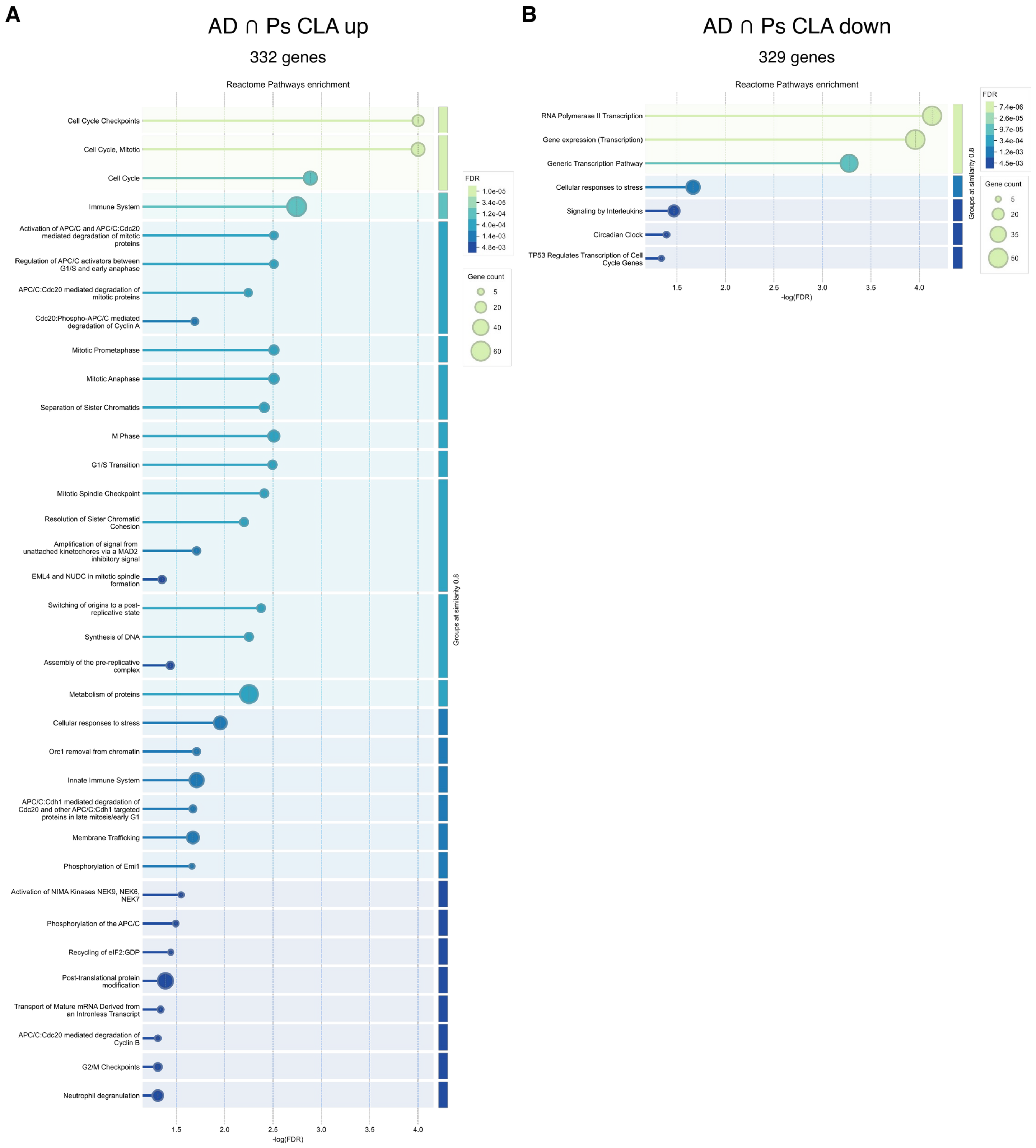

Figure E5. Shared gene expression signature of CD4^{+}CLA^{+} T cells (CLA) from AD and Ps highlights active mitosis. Pathway enrichment analysis was performed using the STRING web tool and Reactome database with shared up- (**A**) or downregulated (**B**) genes.

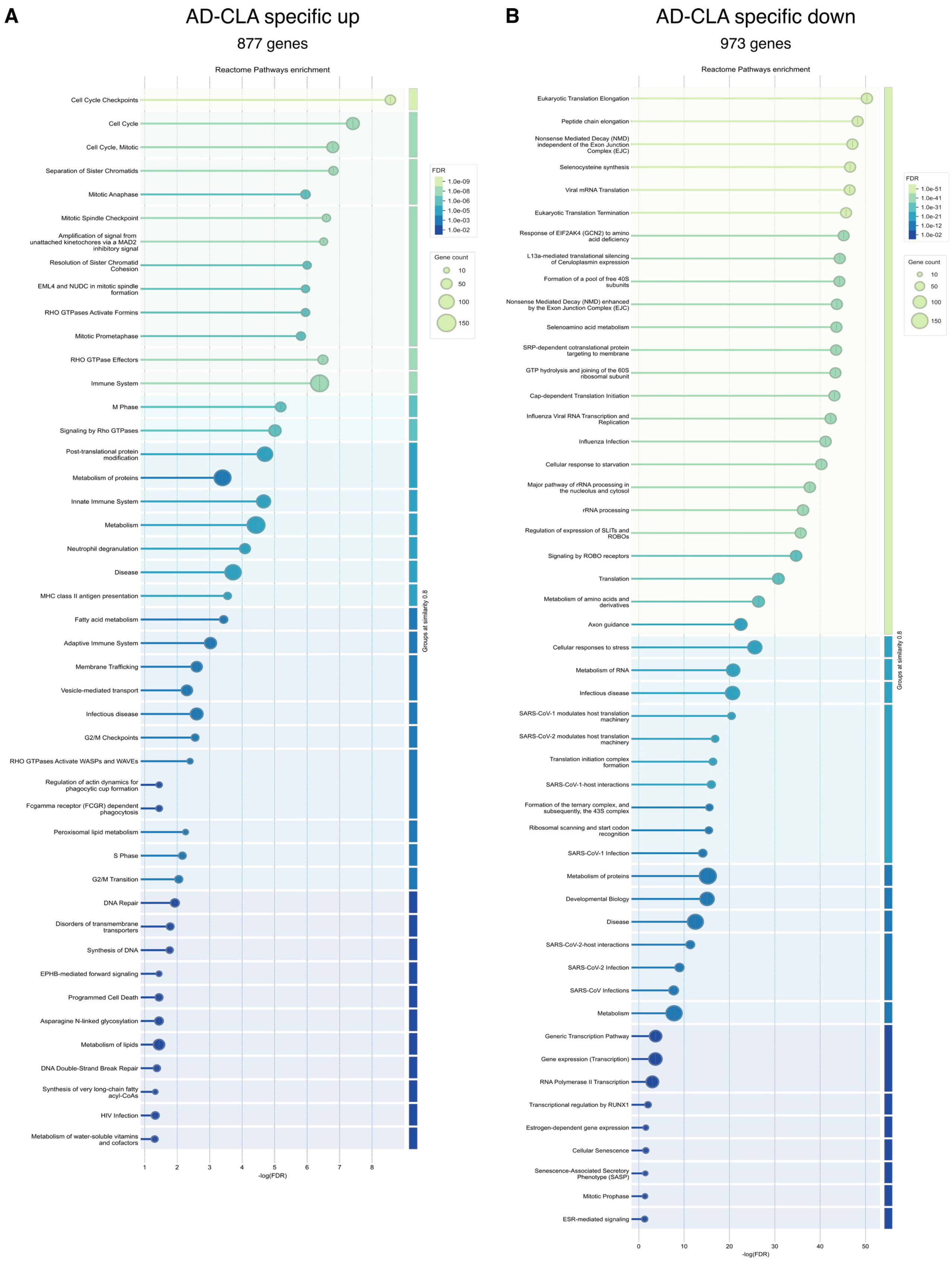

Figure E6. AD specific gene expression signature of CD4^{+}CLA^{+} T cells (CLA) is associated with cell cycle regulation. Pathway enrichment analysis was performed with STRING web tool using Reactome database with AD specific up- (**A**) or downregulated (**B**) genes in CLA cells.

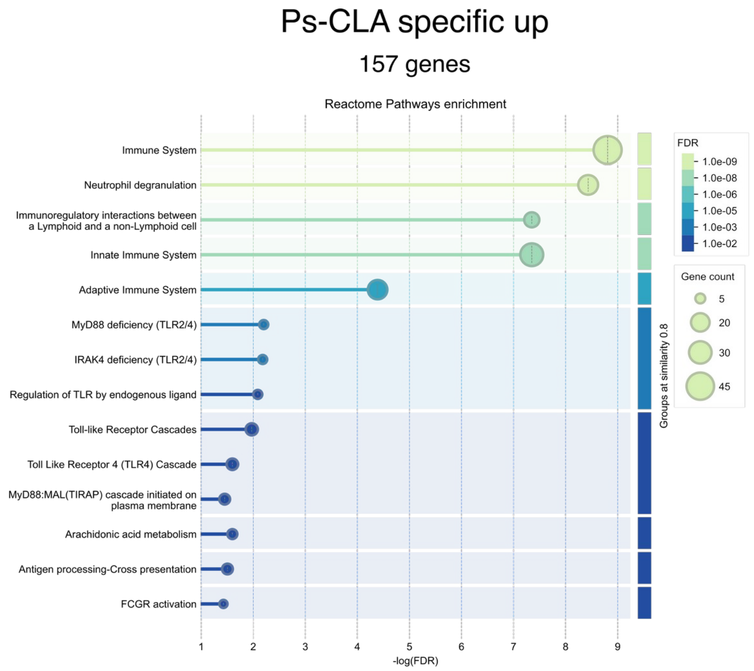

Figure E7. Ps specific gene expression signature of CD4^{+}CLA^{+} T cells (CLA) is associated with innate and adaptive immune system regulation. Pathway enrichment analysis conducted with STRING web tool using Reactome database with Ps specific upregulated genes in CLA cells.

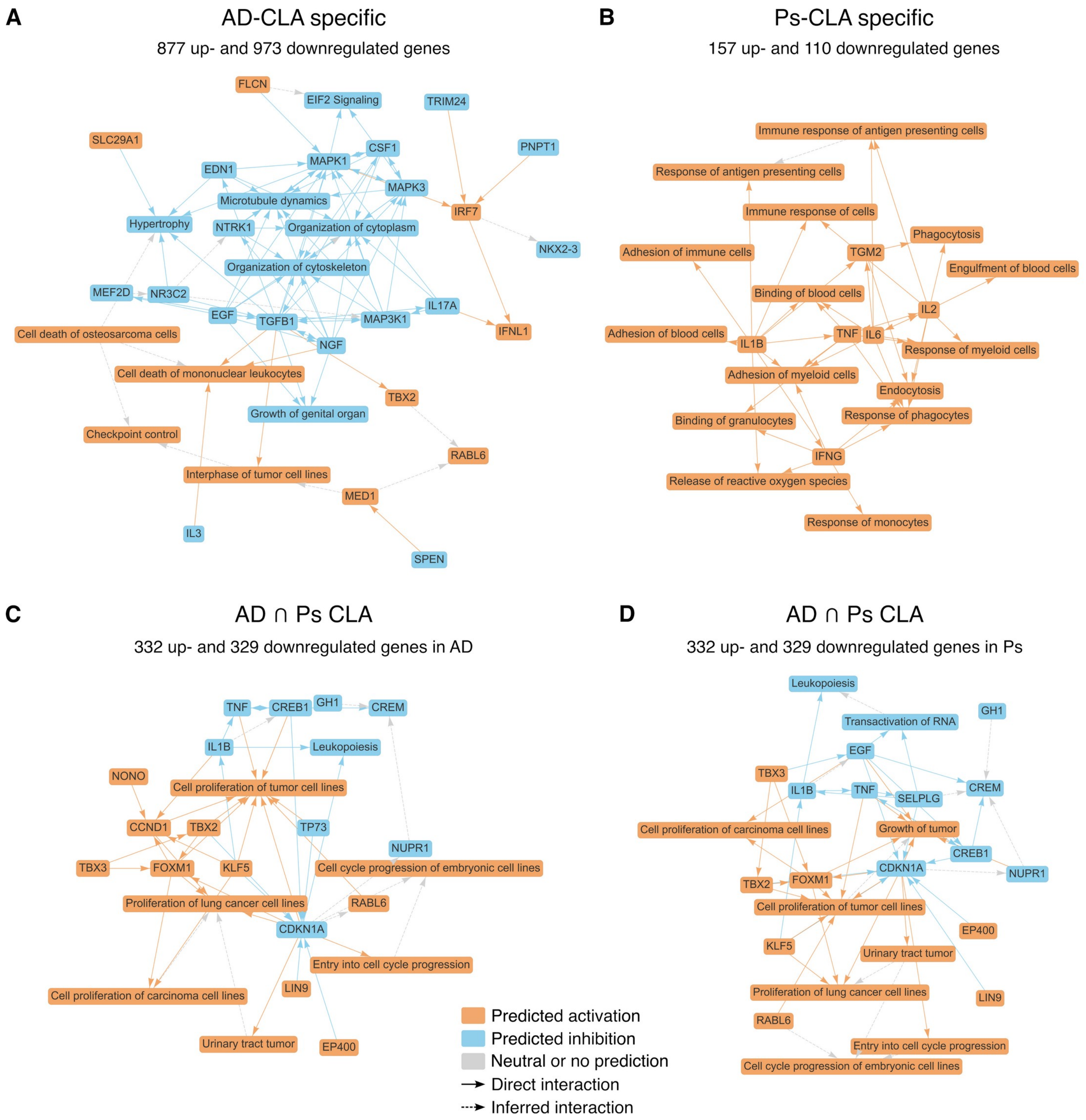

Figure E8. Shared gene expression signatures of $CD4^{+}CLA^{+}$ T cells (CLA) from AD and Ps cells highlight increased proliferation while AD specific signature is associated with cell death regulation and Ps specific with overall activation of innate immune response. IPA of disease specific and shared DE signatures of AD (**A** and **C**) and Ps (**B** and **C**) CLA cells. There are two distinct shared gene expression lists, where expression differences in fold change compared to HC are different for AD and Ps.

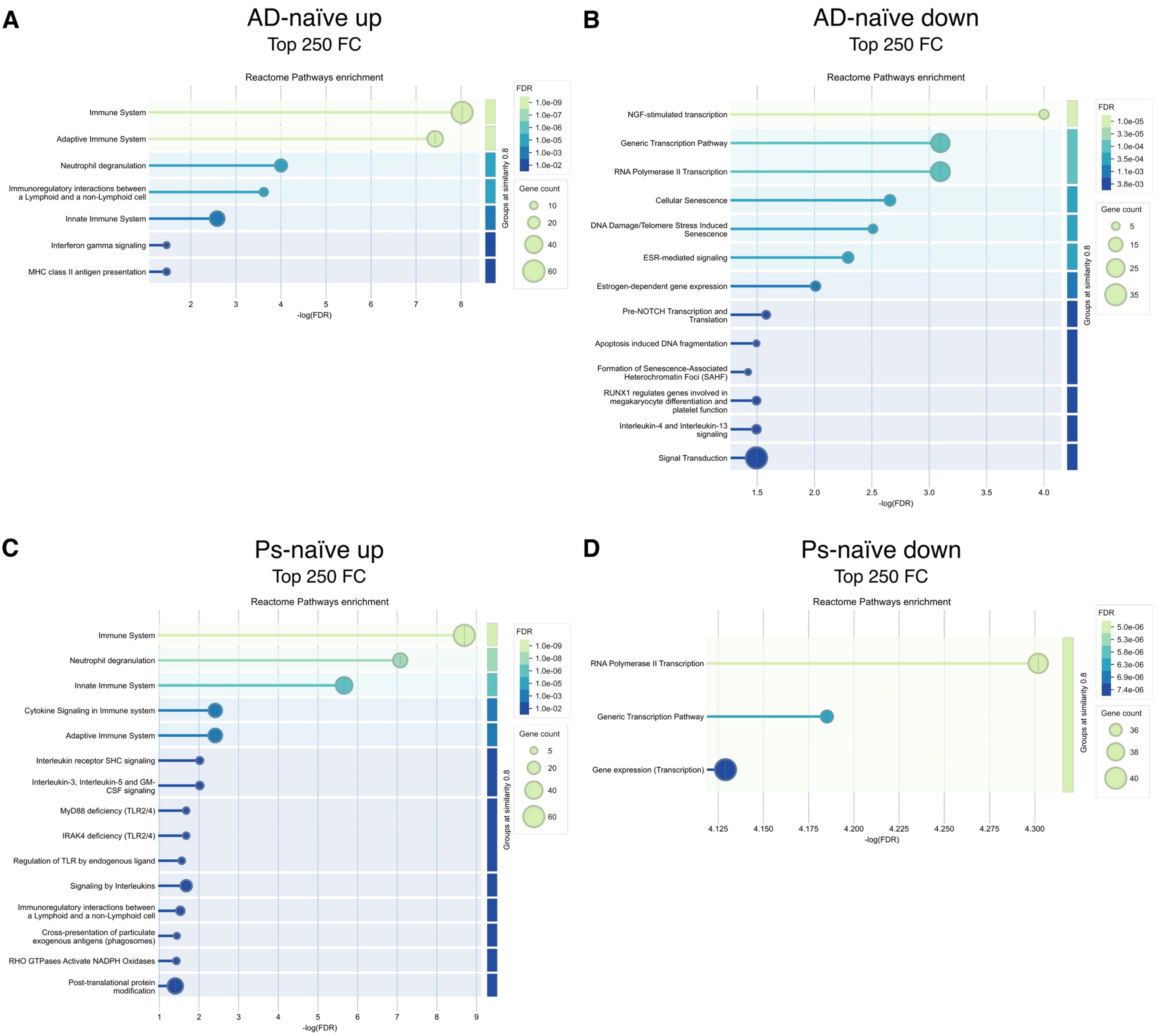

Figure E9. CD4^{+} naïve T cells from AD and Ps show alterations in immune sytem related pathways. Pathway enrichment analysis conducted with STRING web tool using Reactome database with the top 250 most up- or donwregulated genes in AD-naïve (**A** and **B**) and Ps-naïve (**C** and **D**) cells.

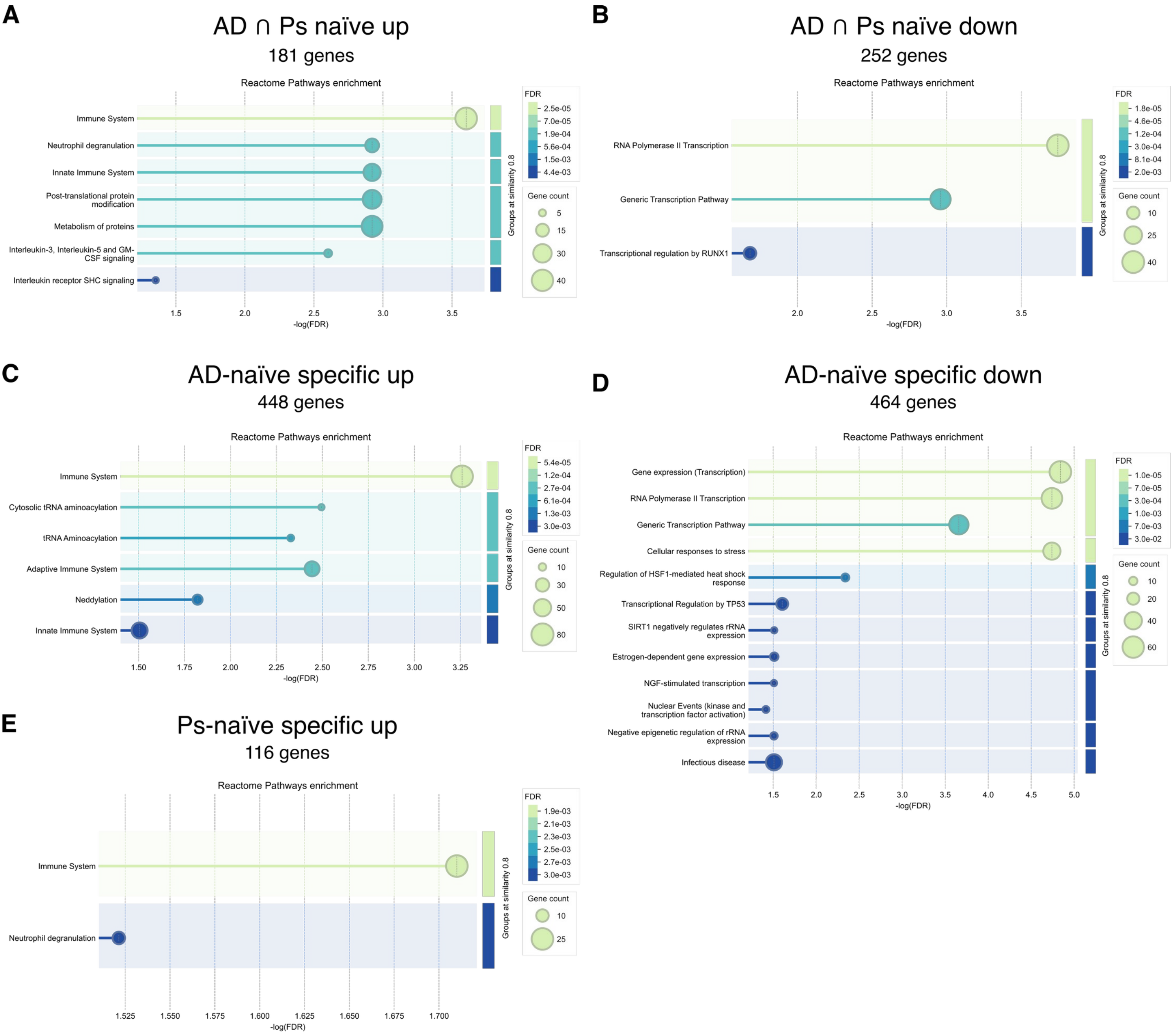

Figure E10. Shared and specific DEGs from CD4^{+} naïve T cells from AD and Ps are associated with immune system related pathways. Pathway enrichment analysis conducted with STRING web tool using Reactome database with shared up- (**A**) or downregulated (**B**) genes and specific upregulated genes in AD (**C**), Ps (**E**) or downregulated genes in AD (**D**).

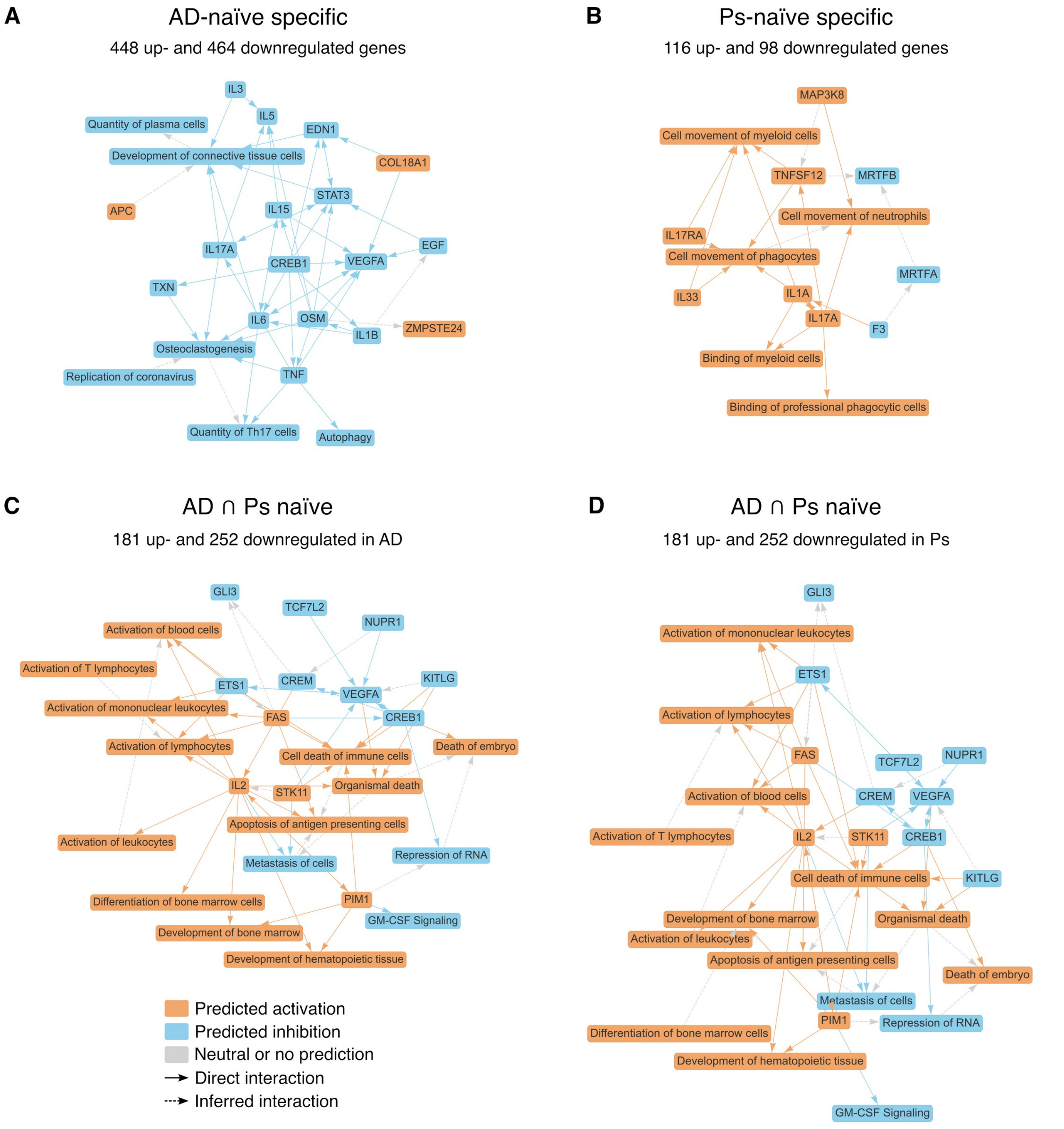

Figure E11. Shared and specific DEGs of CD4^{+} naïve T cells from AD and Ps reveals shared activation involving IL2 and influence of different sets of cytokines for Ps and AD, respectively. IPA of disease specific and shared DE signatures of AD (**A** and **C**) and Ps (**B** and **C**) naïve cells. There are two distinct shared gene expression lists, where expression differences in fold change compared to HC are different for AD and Ps.

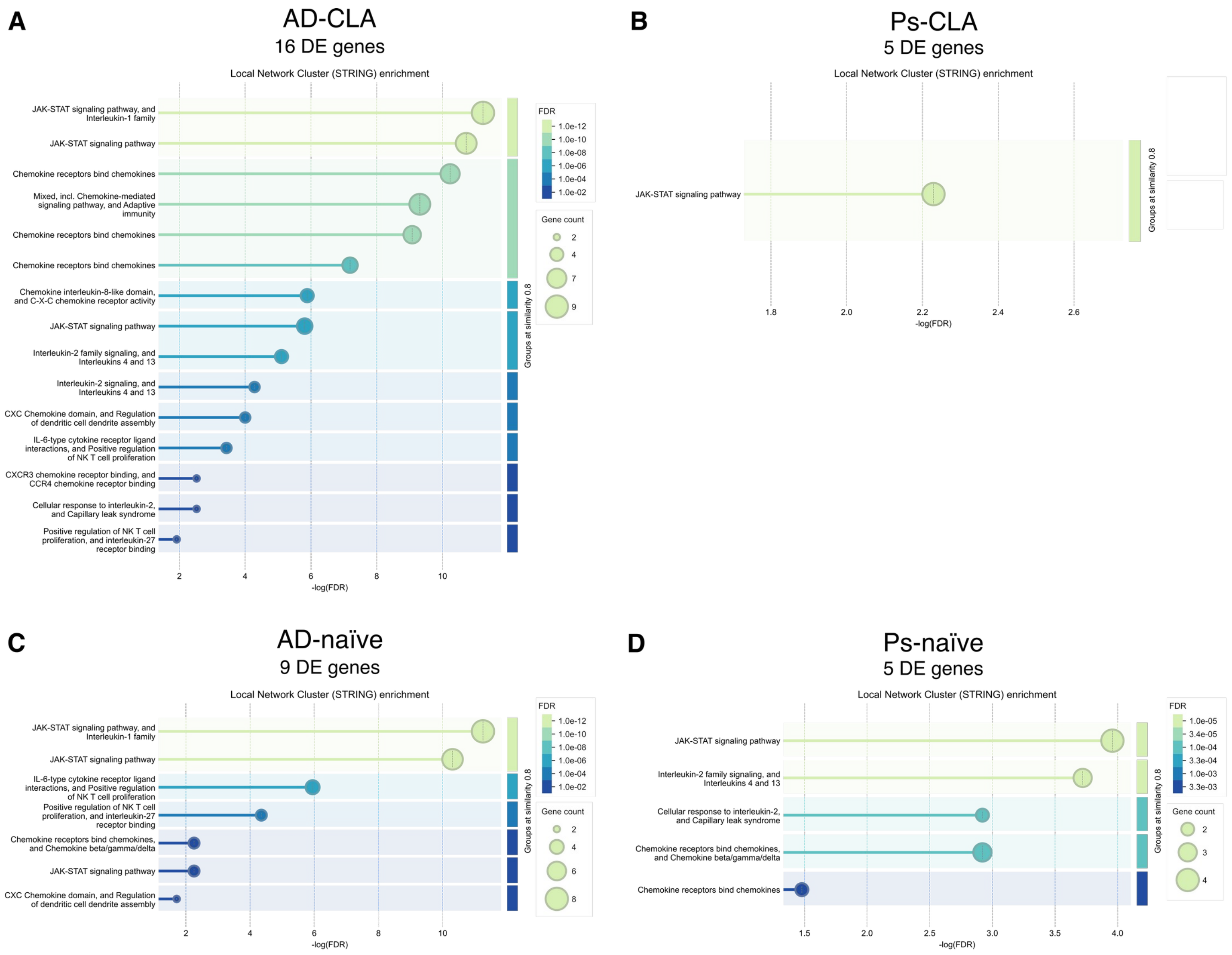

Figure E12. CD4$^+$CLA$^+$ (CLA) or CD4$^+$ naïve T cells (naïve) from AD and Ps have altered cytokine signaling affecting JAK-STAT pathway. Functional enrichment analysis utilizing local STRING network cluster database and DEGs belonging into group of interleukins, chemokines and related receptors (hsa04052 and hsa04050, kegg brite) of CLA and naïve cells from AD (**A** and **C**, respectively) and Ps (**B** and **D**, respectively) with STRING web tool.

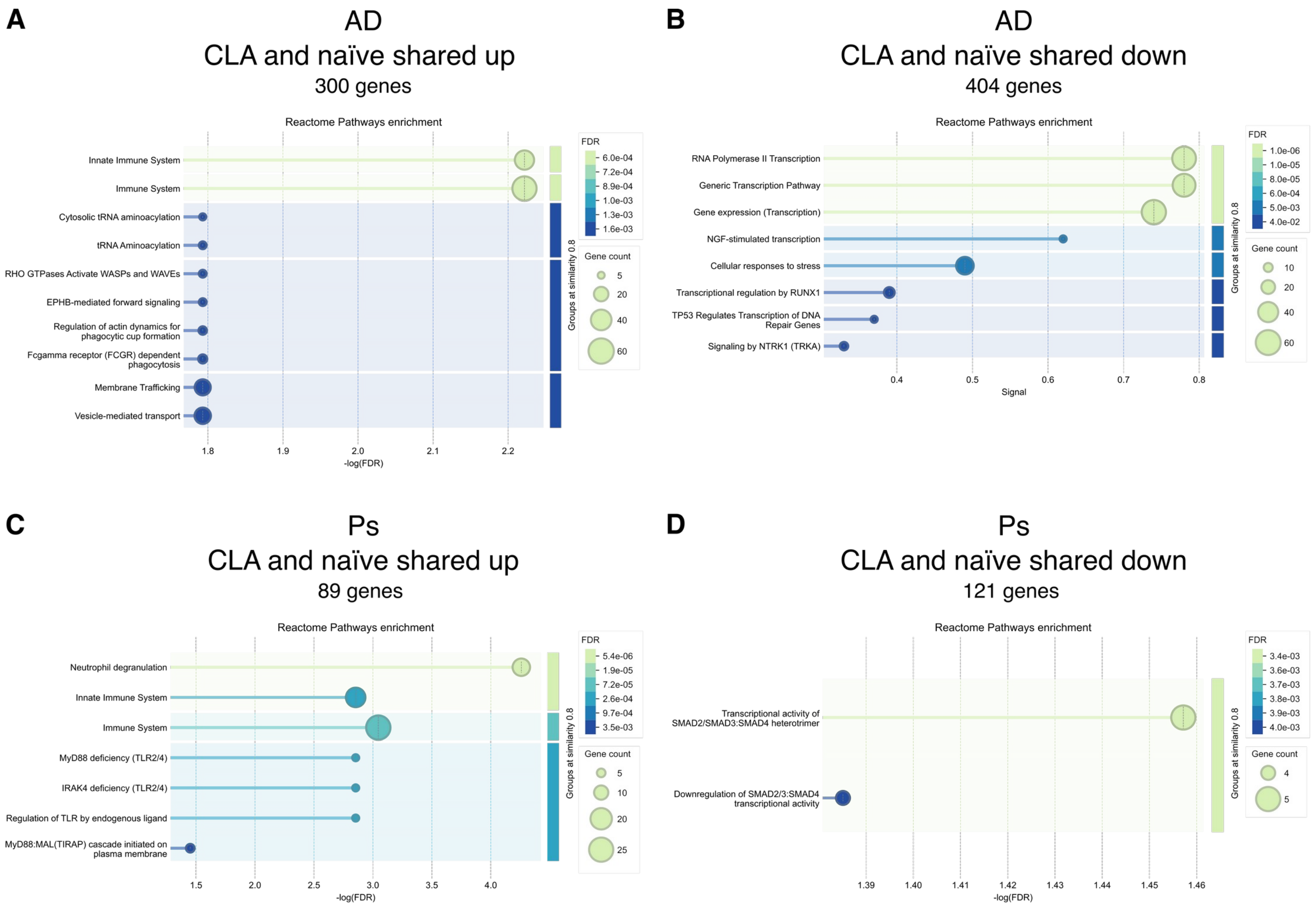

Figure E13. CD4$^+$CLA$^+$ (CLA) and CD4$^+$ naïve (naïve) T cell shared expression signatures. Pathway enrichment analysis conducted with STRING web tool using Reactome database with AD specific up- (A) or downregulated (B) or Ps specific up- (C) or downregulated (D) genes shared in CLA and naïve cells.

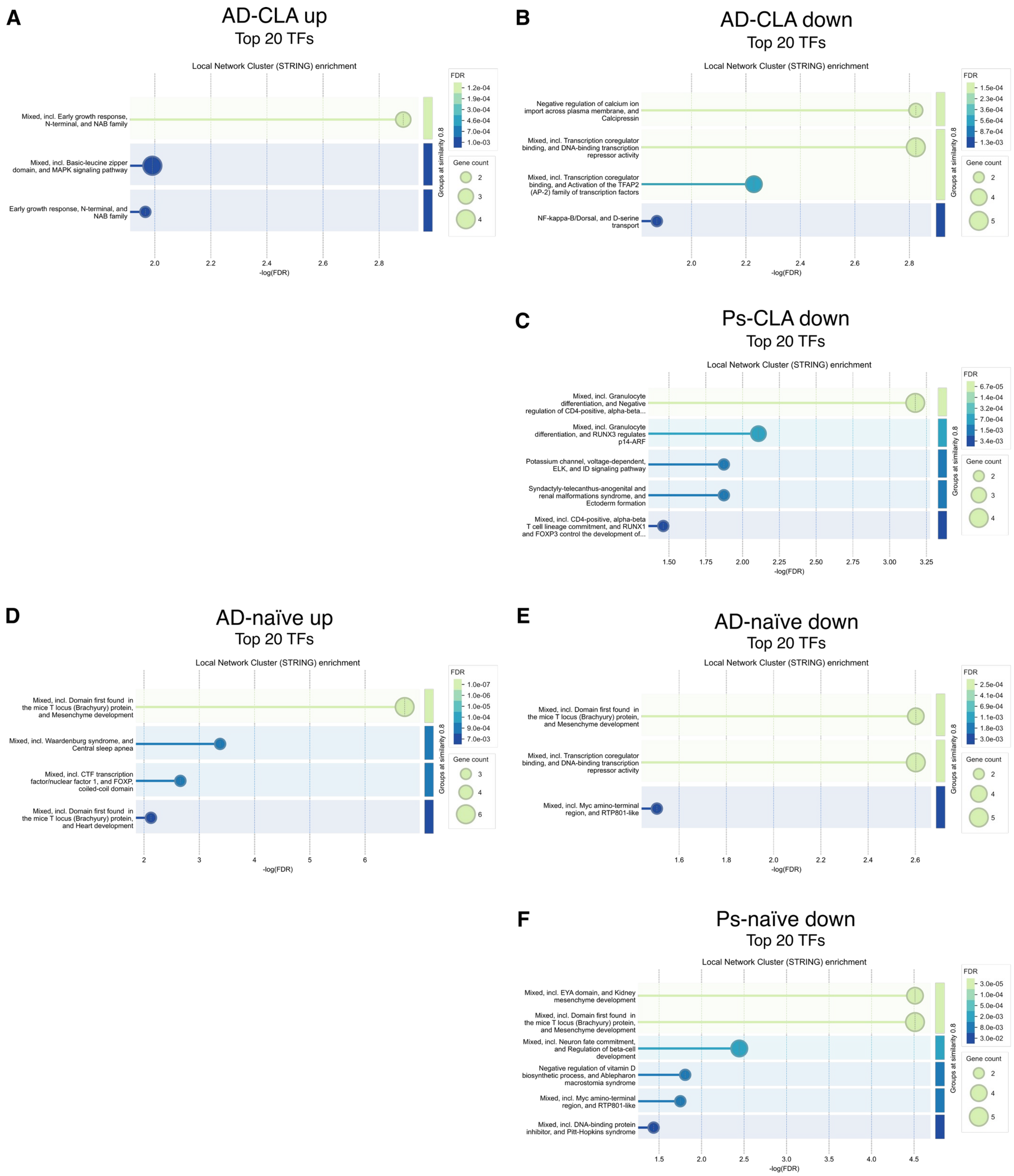

Figure E14. Predicted functionally important TFs in $CD4^{+}CLA^{+}$ (CLA) or $CD4^{+}$ naïve (naïve) T cells from AD and Ps patients. Functional enrichment analysis of the top 20 highest-ranked TFs (predicted by WhichTF) associated with regions of increased (up) or decreased (down) chromatin accessibility, using the local STRING network cluster database. Enriched pathways are presented for CLA cells from AD (**A** and **B**) and Ps (**C**) and naïve cells from AD (**D** and **E**) and Ps (**F**).

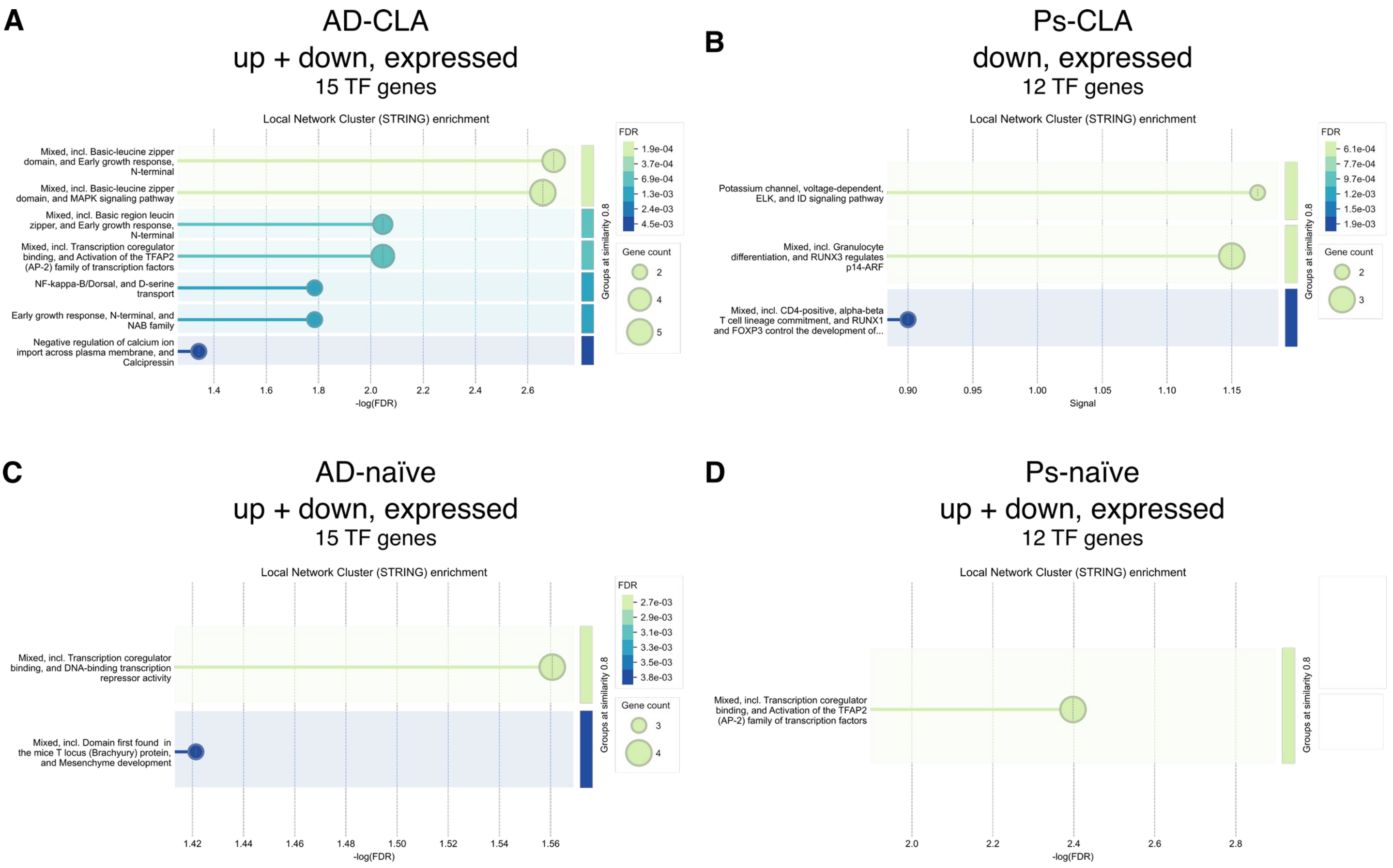

Figure E15. Predicted functionally important TFs expressed in CD4^{+}CLA^{+} (CLA) or CD4^{+} naïve (naïve) T cells from AD and Ps patients. Functional enrichment analysis of the expressed TFs among the top 20 highest-ranked transcription factors (predicted by WhichTF) associated with regions of increased (up) and decreased (down) chromatin accessibility, using the local STRING network cluster database. Enriched pathways are presented for CLA cells from AD (**A**) and Ps (**B**) and naïve cells from AD (**C**) and Ps (**D**).

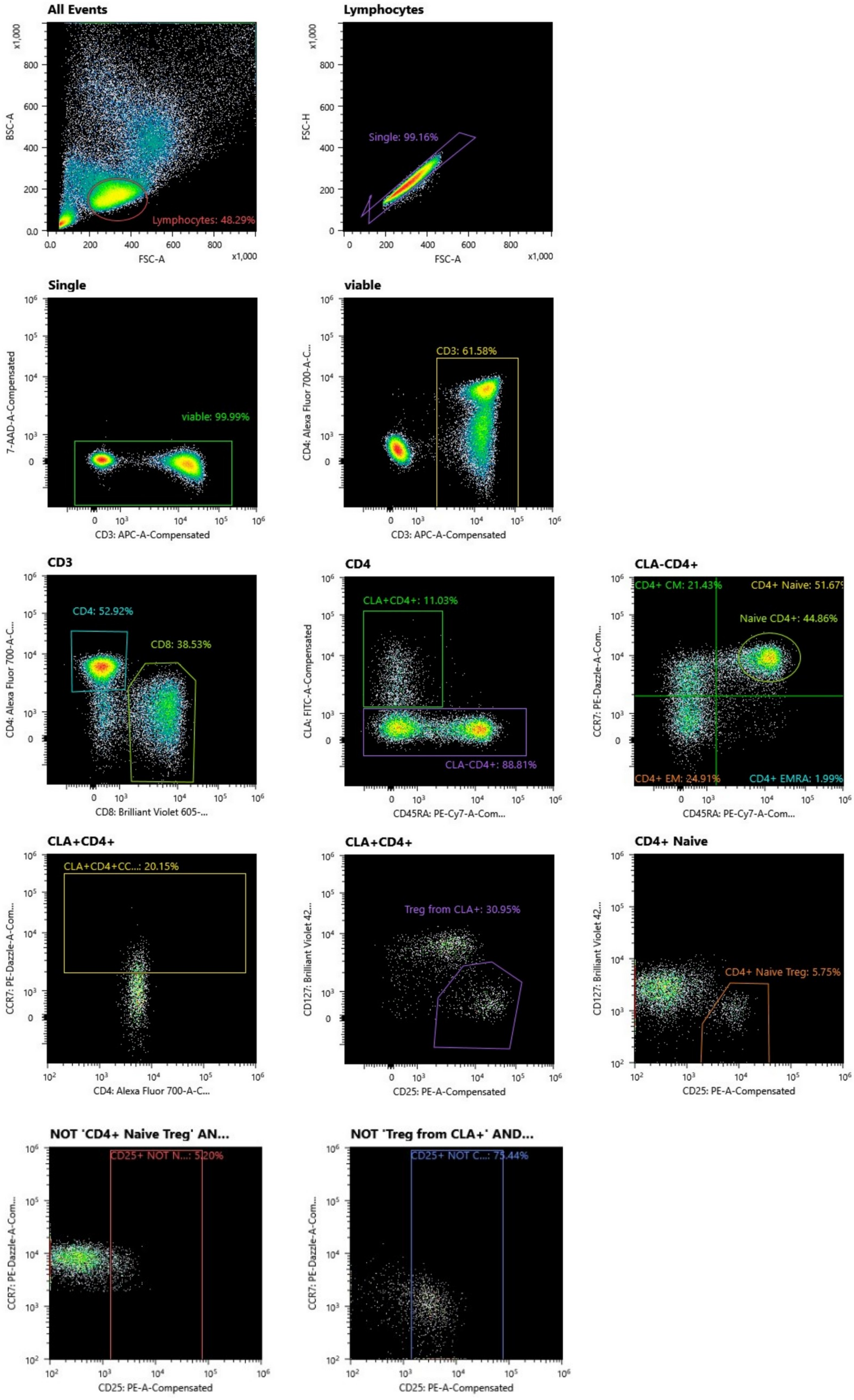

Figure E16. Example of gating strategy for flow cytometric analysis and cell sorting. CD4^{+}CLA^{+} T cells were sorted under gate "CLA^{+}CD4^{+}", while a more conservative gate "Naive CD4^{+}" was used for sorting CD4^{+} naïve T cells. Boolean gates were used to select populations without Tregs for subsequent analysis.

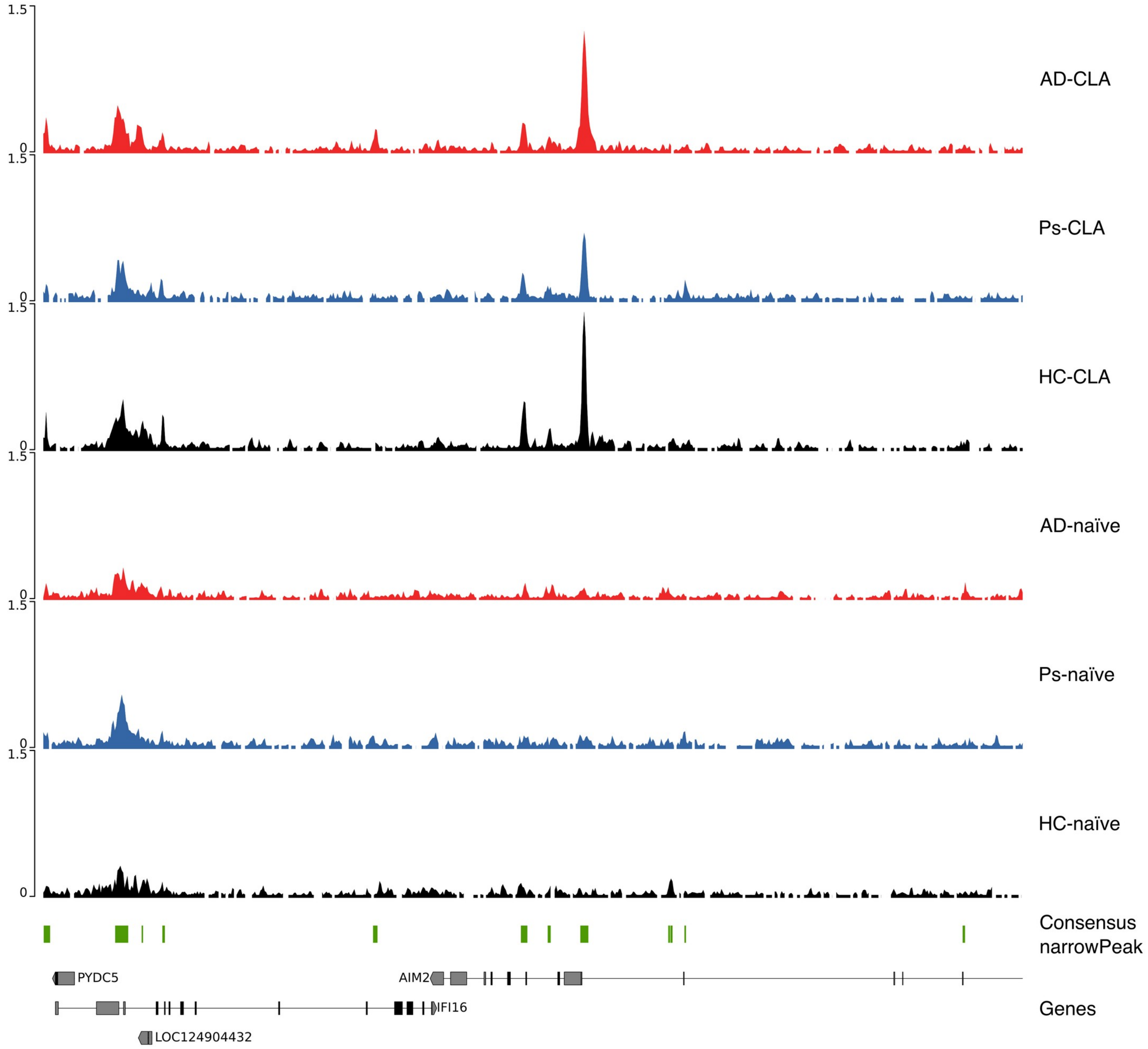

Figure E17. Example of ATAC-seq coverage and peak detection at the *AIM2* gene region in selected patients and healthy controls. The average normalized coverage across genomic bins is shown (summary method: mean; pyGenomeTracks), where the y-axis represents the average chromatin accessibility signal. Regions included in the consensus narrowPeak set are highlighted in green.

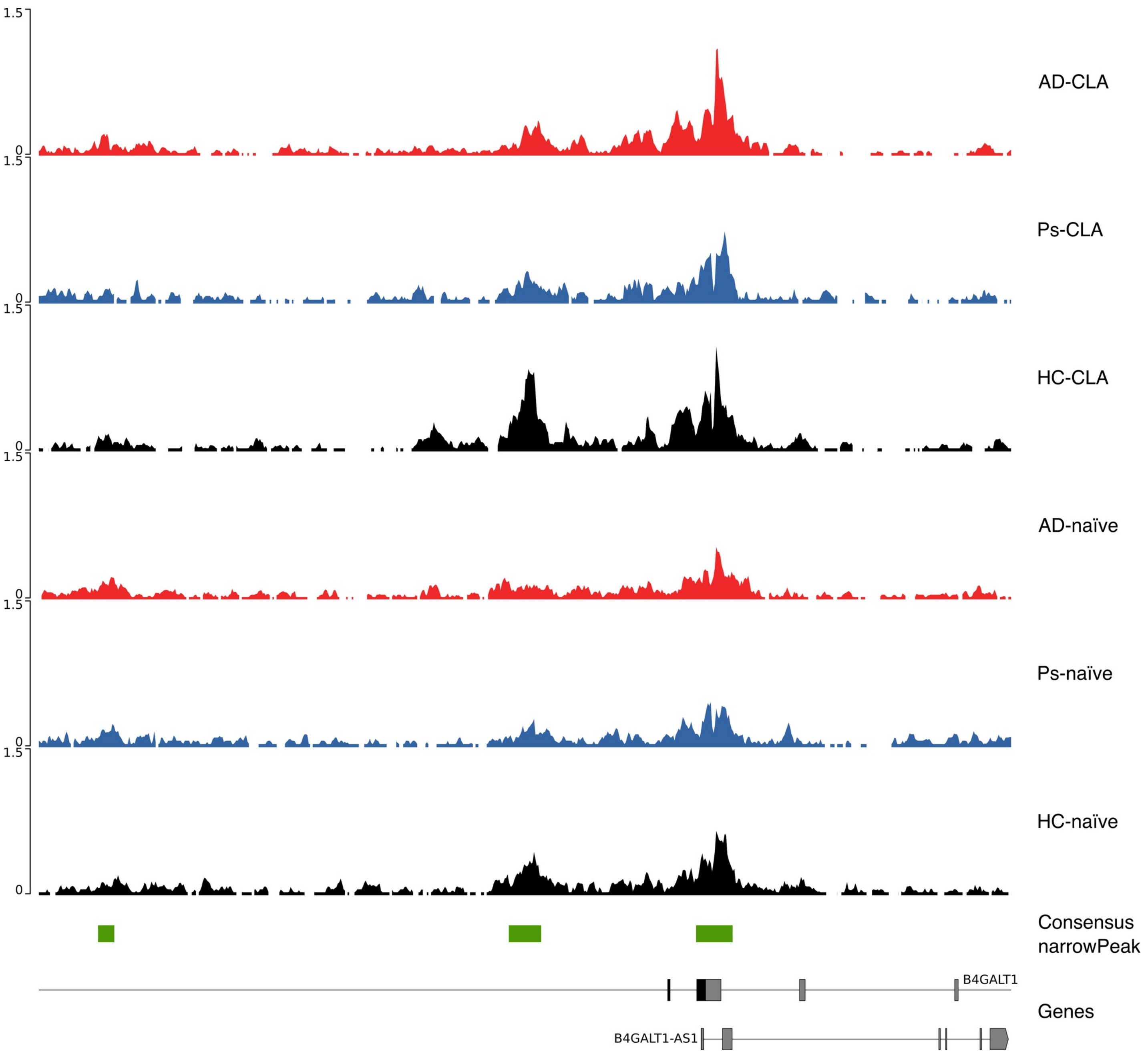

Figure E18. Example of ATAC-seq coverage and peak detection at the *B4GALT1* gene region in selected patients and healthy controls. The average normalized coverage across genomic bins is shown (summary method: mean; pyGenomeTracks), where the y-axis represents the average chromatin accessibility signal. Regions included in the consensus narrowPeak set are highlighted in green.

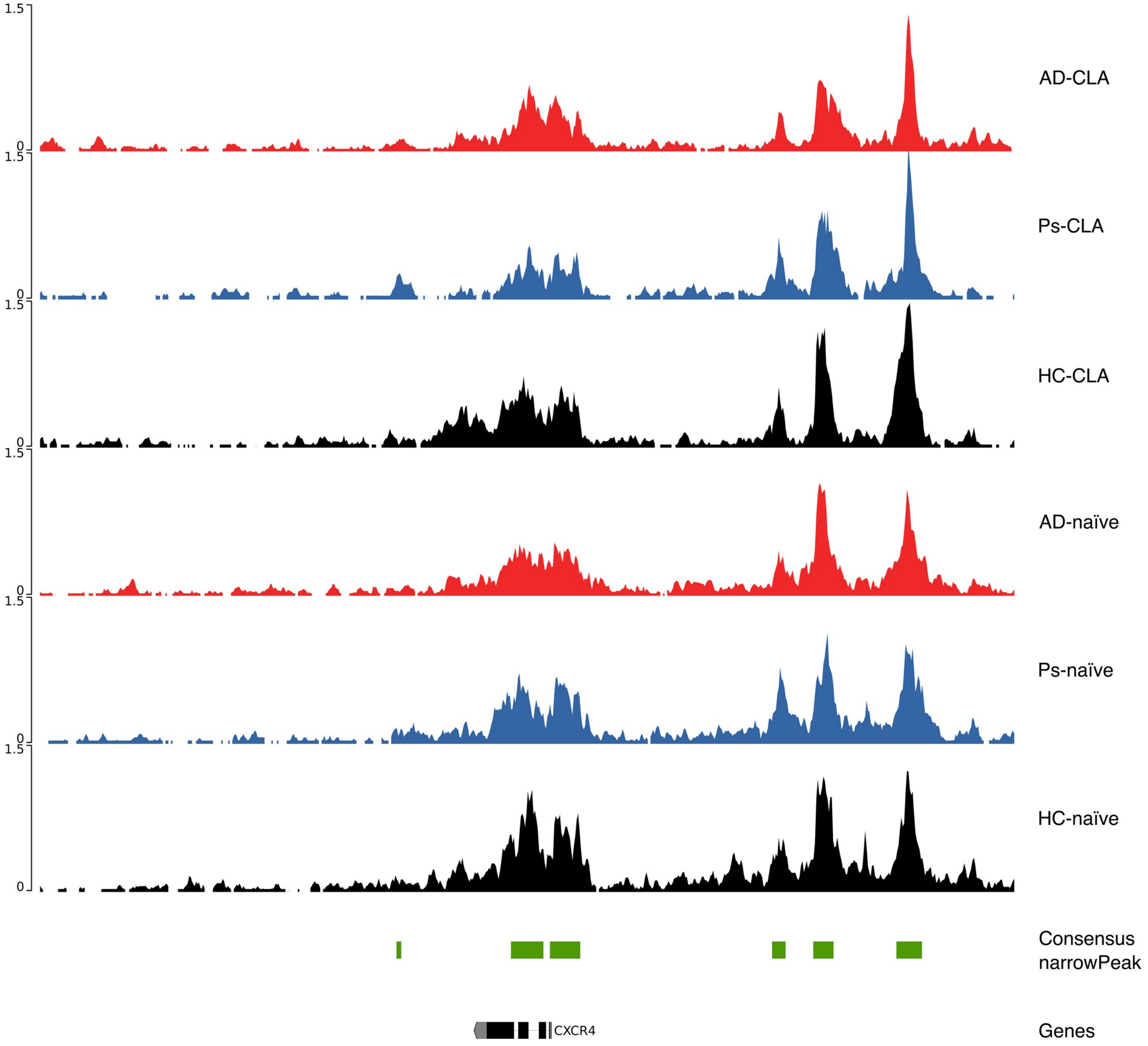

Figure E19. Example of ATAC-seq coverage and peak detection at the *CXCR4* gene region in selected patients and healthy controls. The average normalized coverage across genomic bins is shown (summary method: mean; pyGenomeTracks), where the y-axis represents the average chromatin accessibility signal. Regions included in the consensus narrowPeak set are highlighted in green.
